# Supplementary material for: Nitrogen-15 dynamic nuclear polarization of nicotinamide derivatives in biocompatible solutions
Source: Sci Adv. 2023 Aug 23;9(34):eadd3643. doi: 10.1126/sciadv.add3643 (PMC10446501; doi:10.1126/sciadv.add3643)
Supplement: 20230823-1 [file sciadv.add3643.v1.pdf]

CHEMICAL PHYSICS

Copyright © 2023 The Authors, some rights reserved; exclusive licensee American Association for the Advancement of Science. No claim to original U.S. Government Works. Distributed under a Creative Commons Attribution License 4.0 (CC BY).

Nitrogen-15 dynamic nuclear polarization of nicotinamide derivatives in biocompatible solutions

Josh P. Peters[1], Arne Brahms[2], Vivian Janicaud[1]†, Maria Anikeeva[1], Eva Peschke[1], Frowin Ellermann[1], Arianna Ferrari[1], Dana Hellmold[3], Janka Held-Feindt[3], Na-mi Kim[4], Johannes Meiser[5], Konrad Aden[4,6], Rainer Herges[2], Jan-Bernd Hövener[1]*, Andrey N. Pravdivtsev[1]*

Dissolution dynamic nuclear polarization (dDNP) increases the sensitivity of magnetic resonance imaging by more than 10,000 times, enabling in vivo metabolic imaging to be performed noninvasively in real time. Here, we are developing a group of dDNP polarized tracers based on nicotinamide (NAM). We synthesized 1-^{15}N-NAM and 1-^{15}N nicotinic acid and hyperpolarized them with dDNP, reaching (13.0 ± 1.9)% ^{15}N polarization. We found that the lifetime of hyperpolarized 1-^{15}N-NAM is strongly field- and pH-dependent, with T_1 being as long as 41 s at a pH of 12 and 1 T while as short as a few seconds at neutral pH and fields below 1 T. The remarkably short 1-^{15}N lifetime at low magnetic fields and neutral pH drove us to establish a unique pH neutralization procedure. Using ^{15}N dDNP and an inexpensive rodent imaging probe designed in-house, we acquired a ^{15}N MRI of 1-^{15}N-NAM (previously hyperpolarized for more than an hour) in less than 1 s.

INTRODUCTION

A well-balanced energy metabolism is critical for all living species. For example, glucose metabolism is altered in diabetes (*1*), Pompe disease (*2*), and cancer (*3*, *4*). Detecting metabolic alterations fast, noninvasively, in vivo, and in real time is a promising approach to improve the understanding, diagnosis, and treatment of diseases.

Many efforts have been invested in such imaging techniques. Still, despite all efforts and revolutionary developments in magnetic resonance imaging (MRI) (*5*–*7*), positron emission tomography (*8*), single-photon emission computed tomography (*9*), and fluorescence microscopy (*10*), real-time imaging of in vivo metabolism remains challenging.

Hyperpolarized MRI is the latest addition to this family and has shown ground-breaking results. At standard conditions and clinically relevant magnetic fields, no more than 1 in 100,000 of all nuclear spins contribute effectively to the MR signal. This is due to the low spin polarization at thermal equilibrium and is caused by the energy of the involved spin states, which is much lower than the thermal energy. However, various physical effects (*11*–*14*) have increased the nuclear spin polarization by several orders of magnitude up to unity (from $P \sim 10^{-5}$ to $P \sim 1$) (*13*, *15*). The most advanced technique for polarizing small biomolecules in solution for biomedical applications is dissolution dynamic nuclear polarization (dDNP), which is used here. Leveraging dDNP's advantages, hyperpolarized metabolic ^{13}C-MRI has shown intriguing applications, e.g., for cancer diagnosis, therapy monitoring, and perfusion (*16*–*21*). Other techniques, such as parahydrogen-based hyperpolarization methods, have made great progress but have not reached the maturity needed for clinical studies (*15*, *22*, *23*).

Although the ^{15}N isotope is less common compared to ^{13}C in hyperpolarized MRI, it has very useful properties for metabolic MRI. This includes a relaxation time that is often long (due to its small gyromagnetic ratio, γ) (*24*), a wide range of chemical shift variation, and an abundance in molecules that are central to many metabolic pathways (*25*). At the same time, the low γ causes a low MRI sensitivity and a slow DNP effect (*25*, *26*). Thus, using ^{15}N for hyperpolarized MRI is beneficial when other nuclei are not available, are short-lived, or do not offer sufficient chemical shift (δ) variability (*27*).

Among the nitrogen-carrying biomolecules, nicotinamide (NAM) plays a critical role in glycolysis and the metabolism of fatty acids, cytokines, and other small molecules. NAM is at the crossroads of several metabolic pathways (Fig. 1). Under physiological conditions, intracellular uptake of NAM occurs via binding to a specific plasma membrane site (*28*) and is used to synthesize NAM adenine dinucleotide (NAD). Subsequently, NAD is turned over rapidly due to catabolism via adenosine diphosphate (ADP)–ribosylation and other reactions. This omnipresence of NAM suggests that it can be exploited for diagnosis and in vivo analysis of several enzymatic reactions, particularly as NAM was shown to be safe for in vivo administration (*29*).

More specifically, the first reaction of NAM in vivo is catalyzed by NAM phosphoribosyltransferase (NAMPT). This reaction represents the rate-limiting step for producing NAD from NAM via the salvage pathway (*30*). As NAD$^+$ is essential for glucose metabolism and cancer cells often boost glycolysis, intracellular NAMPT is often amplified in cancer cells (*31*, *32*). The NAMPT (*30*, *33*, *34*) converts NAM to NAM mononucleotide [NMN; 1-^{15}N-NAM(δ_N = 298 ppm) to 1-^{15}N-NMN(δ_N = 219 ppm)], which is converted to NAD$^+$(δ_N = 219 ppm). The metabolite NAM riboside (NR; δ_N = 220 ppm) is closely related to NMN and may be interconverted by exchanging the phosphorus group.

[1]Section Biomedical Imaging, Molecular Imaging North Competence Center (MOIN CC), Department of Radiology and Neuroradiology, University Medical Center Kiel, Kiel University, Am Botanischen Garten 14, 24118 Kiel, Germany. [2]Otto Diels Institute for Organic Chemistry, Kiel University, Otto-Hahn Platz 4, 24098 Kiel, Germany. [3]Department of Neurosurgery, University Medical Center Kiel, Arnold-Heller-Str. 3, House D, 24105 Kiel, Germany. [4]Institute of Clinical Molecular Biology, Kiel University, Rosalind-Franklin-Straße 12, 24105 Kiel, Germany. [5]Cancer Metabolism Group, Department of Cancer Research, Luxembourg Institute of Health, 6A Rue Nicolas-Ernest Barblé, 1210 Luxembourg, Luxembourg. [6]Department of Internal Medicine I, University Medical Center Kiel, Kiel, Germany.
†Present address: Vienna University of Technology, Karlsplatz 13, Vienna 1040, Austria.
*Corresponding author. Email: jan.hoevener@rad.uni-kiel.de (J.-B.H.); andrey.pravdivtsev@rad.uni-kiel.de (A.N.P.)

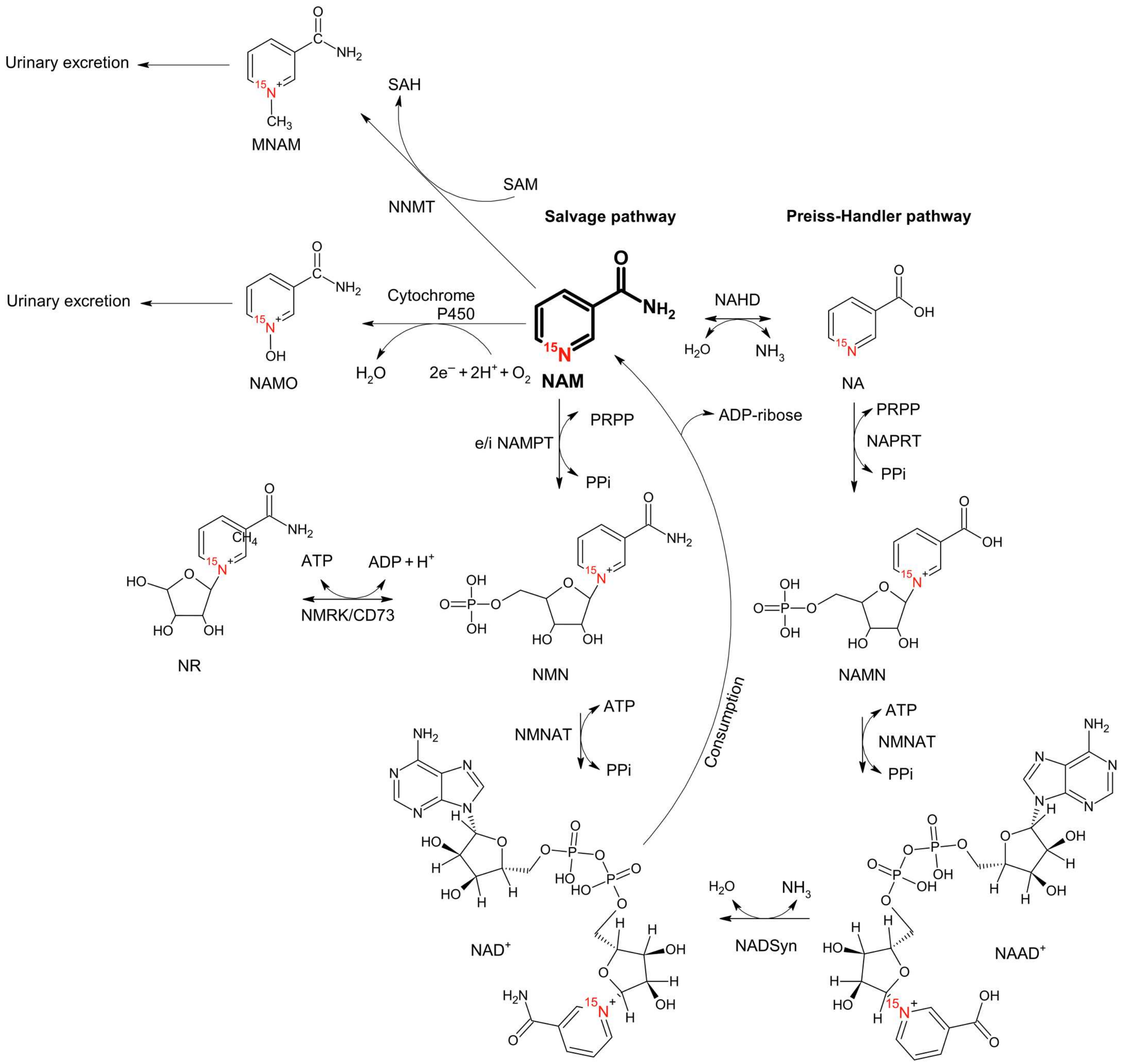

Fig. 1. Metabolic pathways of NAM metabolism. NAM (bold) is at the crossroads of four enzymatic reactions. The salvage pathway describes the re-creation of NAD from NAM, and the consumption of NAD produces NAM as residue (*34*). As an alternative, NR can be produced from NMN instead of NAD (*97*). NAM is mainly excreted through MNAM (*98*) and NAMO (*99*). In addition, NAM can be interconverted to NA by NAHD for the subsequent conversion to NAAD via the Preiss-Handler pathway (*100*). Molecules: NAM, nicotinamide; NA, nicotinate; NMN, NAM mononucleotide; NR, NAM riboside; NAMN, NA mononucleotide; NAMO, NAM *N*-oxide; MNAM, 1-methyl NAM; NAAD, NA adenine dinucleotide; NAD, NAM adenine dinucleotide. All molecules are labeled with ^{15}N in the pyridine ring. Cofactors: SAM, *S*-adenosyl methionine; SAH, *S*-adenosyl-L-homocysteine; PRPP, phosphoribosyl pyrophosphate; Ppi, inorganic pyrophosphate; ADP, adenosine diphosphate; ATP, adenosine triphosphate. Enzymes: NAHD, NAM amidohydrolase; NNMT, NAM *N*-methyltransferase; NAMPT, extra- and intracellular (e/i) NAM phosphoribosyltransferase; NAPRT, NA phosphoribosyltransferase; NMNAT, NAM mononucleotide adenylyltransferase; NADSyn, NA adenine dinucleotide synthetase; NMRK, NAM riboside kinase; CD73, 5′-nucleotidase.

Another NAM pathway is catalyzed by intracellular and extracellular NAM *N*-methyltransferase (NNMT) (*35*) and results in the irreversible conversion of NAM to 1-methyl NAM [MNAM; 1-^{15}N-NAM(δ_N = 298 ppm) to 1-^{15}N-MNAM(δ_N = 203 ppm)]. NNMT is linked to DNA methylation, vitamin metabolism, immune response, and certain types of cancer in the human body and can be found in the fat cells of insulin-resistant animals (*36*).

Other NAM-related enzymes include NAM amidohydrolase (NAHD), which converts NAM to nicotinate [NA; 1-^{15}N-NAM(298 ppm) to 1-^{15}N-NA(296 ppm)], and cytochrome P450, which converts NAM to NAM N-oxide [NAMO; 1-^{15}N-NAM(298 ppm) to 1-^{15}N-NAMO(275 ppm)]. It has been suggested that NAMO is a biological oxidation agent (*37*), while other reactions involving NAM play a less important role (*38*).

Thus, the NAM metabolism appears to be an interesting target for metabolic imaging. Despite these promising properties, there are only a few reports concerning hyperpolarized NAM (and derivatives), none of which addresses biomedical applications. In some examples, ^{1}H and ^{15}N nuclear spins of NAM were hyperpolarized using different methods. Parahydrogen was used for polarizing ^{1}H and ^{15}N of NAM in methanol (*39*–*41*) or ^{1}H of NAM in an aqueous solution (*42*), while for ^{15}N polarization of NAM in an aqueous solution, dDNP was used (*43*). However, no in vivo imaging was reported, and there appeared to be some issues with obtaining high ^{15}N hyperpolarization reliably in an aqueous solution at neutral pH, described here. Throughout this study, we investigated NAM-class molecules for metabolic MRI, including (i) solid-state DNP, (ii) liquid-state polarization after dDNP, (iii) biocompatible formulations, (iv) monitoring of enzymatic conversions with nuclear magnetic resonance (NMR), and (v) fast ^{15}N MRI of NAM in aqueous solution. Several interesting findings emerged, including reliable P(^{15}N) > 10%, ^{15}N hyperpolarization of NAM, NA, and MNAM with dDNP, and a hitherto unknown interplay of pH, magnetic field on relaxation time, and polarization of 1-^{15}N-NAM.

RESULTS

The results are structured into four sections. First, we optimized the composition of the dDNP concentrate such that the highest polarization is achieved in a minimum polarization time. After this, we studied the ^{15}N polarization in a liquid state where the effects of pH and magnetic field on the polarization and T_1 were discovered. Then, we studied exemplary enzymatic and chemically induced conversions of NAM. In the last section, we demonstrate the feasibility of ^{15}N MRI in an aqueous solution.

Solid-state polarization of 1-^{15}N-NAM

The first step was the optimization of the solid-state ^{15}N DNP polarization, which was essential for all parts of the study. We varied the sample composition by the amounts of 1-^{15}N-NAM, distilled water, glassy agent (glycerol or trehalose), relaxation agent [Gd] (gadobutrol Gd-DO3A-butrol), and radicals (trityl AH111501, trityl OX063, and nitroxyl TEMPO). We carried out three to six dDNP experiments with each composition to find the optimum (compositions are detailed in table S1).

Early on, we found that a neutral pH of the final aqueous solution did not result in any observable liquid-state polarization 17 to 20 s after dissolution, although a high solid-state signal was detected (Fig. 2). Experiments with dissolution media (DMs) that led to a final sample with acidic (pH 0.5 to 2, acidic DM), neutral (pH 7.5 to 8, neutral DM), and basic (pH 12 to 13, basic DM) pH revealed that only the basic DM yielded reliable liquid-state 1-^{15}N signal. Therefore, basic DM was used at the optimization stage. This pH effect is discussed in more detail later.

Choice of glassing agent

A glassy sample is needed for efficient propagation of the polarization within the sample during DNP. As a powder, NAM does not form a glassy matrix on its own and thus must be dissolved in water with the addition of a glassing agent. Glycerol and trehalose are two agents that were used previously as DNP glassing agents (*44*). Trehalose, in contrast to glycerol, does not affect metabolism, requires a lower concentration, and tolerates higher temperatures for matrix formation (*44*, *45*). In addition, we found that trehalose allowed to dissolve more NAM and resulted in a less viscous concentrate compared to glycerol, which was advantageous in preparing the sample and placing it into the vial for DNP. Trehalose concentrations of 0.7 to 0.8 M yielded sufficient glassiness of the sample with up to 3.9 M NAM. Dimethyl sulfoxide (DMSO), another common glassy agent, was discarded because its usability in vivo is limited.

Choice of radical

The radicals are the source of spin order in DNP experiments, as the thermal polarization of the unpaired electron spin approaches 100% at 6.7 T and 1.2 K. The amount and type of radical greatly affect the speed and final level of the DNP (*46*). All radicals tested here (trityl AH111501, trityl OX063, and nitroxyl TEMPO) yielded hyperpolarized, solid-state ^{15}N NMR signals. This solid-state signal was used to adjust the parameters of microwave (MW) irradiation, which drives the polarization transfer between the electrons and nuclei (^{15}N). Unfortunately, the sensitivity was insufficient for detecting solid-state ^{15}N signal in thermal equilibrium, preventing us from quantifying the solid-state polarization. Instead, we used the ^{15}N liquid-state polarization after dissolution with basic DM.

We found that for trityl AH11150, a radical concentration of ca. 29 mM (tested regions, 13.8 to 40.5 mM) yielded the highest liquid-state polarization (Fig. 2; ^{15}N polarization buildup time constant, $T_{\text{b-up}}$, was 1.1 hours, and solid state T_1 was 25.8 hours for [AH111501] ~ 29 mM). The time constant of the ^{15}N polarization buildup varied strongly with the radical concentration, e.g., $T_{\text{b-up}}$, = 0.5 hours for [AH111501] = 40.5 mM to 9 hours for 13.8 mM. Neither polarization buildup rate nor liquid-state polarization was improved when OX063 was used; the liquid-state polarization with TEMPO was about 10 times lower. Therefore, AH111501 and OX063 appear to be good choices for dDNP of ^{15}N-NAM at 6.7 T, and AH111501 was used henceforth because of better availability.

Improving DNP by adding gadolinium

Adding relaxants (e.g., gadolinium) to the solid-state sample was shown to improve the solid-state buildup rates and final polarization (*47*–*50*). Therefore, we investigated the effect of [Gd] on the polarization of 1-^{15}N-NAM. We found that the solid-state polarization increased faster and reached a higher value when up to ~1 mM [Gd] was added. Unfortunately, concentrations larger than 0.5 mM reduced the liquid-state ^{15}N T_1 relaxation time from T_1(0 mM) = 52 s to T_1(1 mM) = 42 s and T_1(2 mM) = 26 s at 1 T. Only a very little signal could be observed in the solid and liquid states for [Gd] = 5 mM, and T_1 was as short as 11 s at 9.4 T; owing to the low signal, we could not observe the signal at 1 T. As 0.5 mM [Gd] resulted in a faster (~14%) and higher (up to 100%) solid-state polarization, we chose this concentration for some of the following experiments.

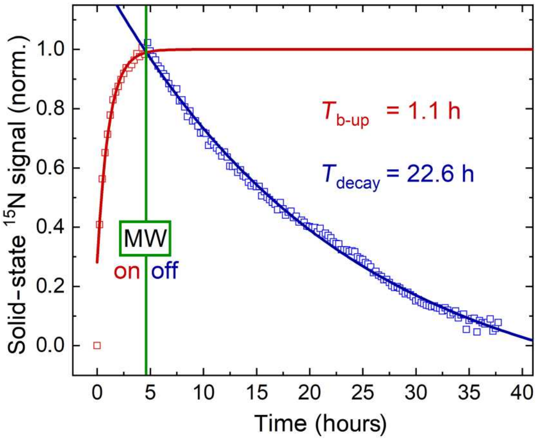

Fig. 2. Solid-state ^{15}N polarization buildup and decay after MW irradiation at 6.7 T and 1.4 K. The polarization buildup ($T_{b\text{-}up}$, red squares) was monitored for ≈5 hours during DNP and the signal decay (T_{decay}, blue squares) for 30 hours thereafter using a 3° RF excitation every 15 min. A monoexponential fit (solid lines) yielded a buildup time of 1.1 hours (red line) and a solid-state polarization decay of 25.8 or 22.6 hours, whether corrected for excitations or not (Eq. 3).

To summarize, the following sample composition promises good ^{15}N dDNP: around 100 mg of 1-^{15}N-NAM, 35 to 70 mg of trehalose, 0 to 0.5 mM [Gd], 9 to 11 mg of AH111501, and 110 to 130 mg of water. This composition gives the following concentrations before dissolution: 3.3 to 3.7 M 1-^{15}N-NAM, 0.5 to 1 M trehalose, 0 to 0.5 mM [Gd], 28.8 to 30.6 mM AH111501, and 0.45 to 0.6 M water.

Liquid-state polarization of 1-^{15}N-NAM after the dissolution

The highest ^{15}N polarization of 1-^{15}N-NAM of (13.0 ± 1.9)% was measured around 20 s after dissolving the sample in 4 ml of basic DM (9.4 T, N = 3; Fig. 3A, spectrum 1a). The dDNP sample composition was as follows: [1-^{15}N-NAM] = 3.4 M, [AH111501] = 29 mM, [H_2O] = 0.45 M, [trehalose] = 0.83 M, and [Gd] = 0.5 mM (liquid-state concentrations, except water, were about 100-fold lower due to dissolution of 55-mg sample with 4-ml DM). The polarization levels for other sample compositions are detailed in table S1.

Impact on the liquid-state hyperpolarization lifetime of 1-^{15}N-NAM

Once dissolved, a reasonable hyperpolarization lifetime (T_1) of the tracer is needed for preserving polarization and observing metabolism. We found that the lifetime of hyperpolarized 1-^{15}N-NAM in basic DM depended on B_0, with the highest value of (41.2 ± 7.2) s recorded at 1 T [Fig. 3B; T_1(0.57 T) = (33.7 ± 8.4) s, T_1(7 T) = (42.4 ± 0.8) s, and T_1(9.4 T) = (28.2 ± 1.7) s]. We attribute the decrease in T_1 at 9.4 T to chemical shift anisotropy (*51*).

We tested several other concentrations and volumes to investigate the varying T_1, especially for lower fields. We found that T_1 was affected by the volume of the DMs: 4 ml of basic DM yielded T_1 = (35.8 ± 3.8) s, while 8 ml yielded (55.9 ± 2.5) s (fig. S4). Note that we carried out experiments with different amounts of DM while keeping the final concentrations of 1-^{15}N-NAM and trityl radical after dissolution constant. Similar results were observed when we kept the amount of the DNP sample constant while doubling the volume of DM (resulting in halved concentrations). Thus, the varying T_1 cannot be explained by concentrations of [radical], [1-^{15}N-NAM], or possible impurities from the chemical synthesis. Instead, we attribute this effect tentatively to different filling levels of the heating chamber and corrosion therein (so that corrosion products dissolve in the DM during heating). Visual inspection of the heating chamber supported this hypothesis.

Liquid-state hyperpolarization at neutral pH

Unfortunately, neutral DM yielded no observable polarization of 1-^{15}N-NAM (Fig. 3A, spectrum 4) and neither did deuterated water, filtering the radical, nor transportation in a 0.2- to 1-T transfer magnet (fig. S15). Thus, we suspected that the temperatures, fields, and pH occurring during transfer shorten T_1 drastically.

We measured T_1 at pH 7.37 and 9.4 T and found an approximately linearly increasing 1-^{15}N-NAM T_1 between 273 and 340 K from 10 to 40 s (fig. S8)—values that should be long enough for the signal to survive and possibly a metabolic application. However, we expect that T_1 is much different at the Earth's field (and others) experienced during transfer compared to 9.4 T. An estimation of T_1 in the stray field of the 9.4-T magnet yielded a T_1 of 2.5 s and may explain the matter (detailed below). Measuring T_1 as a function of the pH of the DM in the range from 1 to 13 yielded quite interesting results: No signal was observed between pH 4 and 6, but T_1 increased steeply outside this range to $T_1 \approx 25$ s at pH 8 to 9 and the highest values of 40 to 45 s at pH 13 (fig. S5).

The radical is a potential cause of fast relaxation and loss of polarization. Using acidic DM, we precipitated trityl radical and filtered it (*13*) right after dissolution; again, no 1-^{15}N-NAM signal was observed at 1 or 9.4 T at acidic pH independent of radical filtering. As neither the concentration nor filtering of the radical affected the liquid state T_1 substantially, we refrained from filtering the radical (table S2). Note that for preclinical examinations, trityl radical at similar concentrations is not typically removed as its toxicity lies within acceptable limits (*52*, *53*).

To advance the matter, we focused on transferring the solution at basic pH and neutralizing it shortly before detection. First attempts of neutralizing the solution by adding HCl to an NMR tube resulted in two distinct phases (clear and milky), which were visually apparent and yielded two different resonances (Fig. 3A, spectrum 2, and fig. S6) that corresponded to the signals below and above pK_a (where K_a is the acid dissociation constant) of NAM of 3.35 (*43*, *54*). Some precipitation of NAM (and likely radical) was observed. Thus, strong shaking (or mixing) was essential to obtain a homogeneous solution with this approach. Vigorous shaking for ~4 s resolved this matter, but the polarization was reduced by 80%. By holding the sample above the magnet after shaking, we estimated the relaxation time of 1-^{15}N-NAM at neutral pH in the stray field to T_1 = 4 s/ln(0.2) = 2.5 s, explaining the complete loss observed during 20-s transfer at neutral pH.

In the end, the following protocol emerged from observing hyperpolarized 1-^{15}N-NAM at neutral pH:

1) Perform dissolution with basic DM;

2) Add 500 μl of the hyperpolarized 1-^{15}N-NAM to the NMR tube;

3) Transfer at pH ~ 12 with or without transfer magnet (0.5 to 1 T) into the fringe field of the detection system (20 to 30 s);

4) Add ~10 μl of concentrated HCl to the NMR tube right in the stray field, above the vertical-bore, high-resolution NMR (or benchtop NMR);

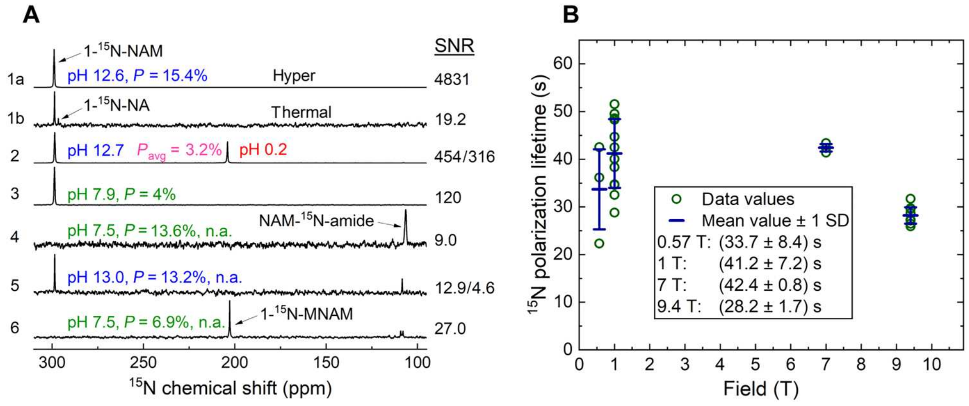

Fig. 3. Effect of pH on liquid-state hyperpolarization of NAM and MNAM at 9.4 T and relaxation time of NAM in 0.57- to 9.4-T range of magnetic fields. (**A**) A strong hyperpolarized 1-^{15}N-NAM signal was observed at basic pH and quantified to ≈15% (1a and 1b). Adding the basic solution to a buffer inside the NMR tube with insufficient sample mixing resulted in two phases with different pH and different 1-^{15}N-NAM resonances with an average polarization of 3.2% (2). When the sample was more rigorously (and longer) mixed, a neutral pH solution with a much-reduced polarization of 1-^{15}N-NAM 0.4% was achieved (3). Repeating the experiment with natural abundance NAM in deuterated neutral DM showed no 1-^{15}N polarization but strong ^{15}N-amide polarization of 13.6% (4), while using protonated basic DM yielded both 1-^{15}N and ^{15}N-amide resonances well polarized (5). A promising agent is MNAM which yielded high polarization ≈ 7% in neutral DM (6): T_1 was short for ^{15}N-amide (≈7.2 s) and long for 1-^{15}N-MNAM (85.1 s). Spectra were obtained 17 to 21 s after dissolution without neutralization and up to 30 s with neutralization. For (1b), 64 transients were acquired using a 90° FA, 101 linear receiver gain (RG). For (6), one transient was acquired using a 10° FA and RG of 101. All other spectra were acquired using 5° FA, RG of 10 in a single scan that should be considered for calculating polarization. (**B**) Lower magnetic fields (down to 1 T) resulted in a longer liquid-state hyperpolarization: T_1(0.57 T) = (33.7 ± 8.4) s, T_1(1 T) = (41.2 ± 7.2) s, T_1(7 T) = (42.4 ± 0.8) s, and T_1(9.4 T) = (28.2 ± 1.7) s. Variation in T_1 was higher at lower fields, which we attributed to varying amounts of paramagnetic impurities or pH.

5) Vigorously shake the sample tube (approximately 4 s); and
6) Place the sample in the isocenter to observe the NMR signal.

Further optimization of the procedure (e.g., neutralizing at high field or within the bore of the MRI) may improve the polarization. Using our low-field systems and the above-described neutralization protocol, we found that T_1 at around pH 7 and 1 T is only 7 to 12 s and 11 to 17 s at 9.4 T—much shorter than at basic pH.

In view of these results, the 1-^{15}N nucleus of NAM appears to be a challenging choice for hyperpolarized MRI. Typically, the use of deuterated dissolution solvent improves the relaxation lifetime and, hence, polarization. However, we did not observe any 1-^{15}N-NAM signals in this case, even when deuterated neutral DM was used. Contrary to common expectations, T_1 and polarization values were even lower when deuterated basic DM was used.

To verify that we did not introduce any impurities, leading to fast relaxation at neutral pH, during the synthesis of 1-^{15}N-NAM, we hyperpolarized natural abundance NAM for over 14 hours. As the ^{15}N signal was about 300-fold lower (0.3% natural abundance), no solid-state ^{15}N signal was observed during polarization buildup, and we used the dDNP parameters optimized for 1-^{15}N-NAM. After dissolution with basic DM, the sample was measured. Similar T_1 and polarization values were obtained (Fig. 3A, spectrum 5).

The second nitrogen of the amide group appears to be less affected by the T_1 issue described above. For example, the maximum polarization in deuterated neutral DM was quite high at ~13.6% (Fig. 3A, spectrum 4, and fig. S3). The lifetime of polarization at 9.4-T deuterated neutral DM was T_1(NAM-^{15}N-amide) = 58 s and in deuterated basic DM was 45.5 s, which is longer than T_1(1-^{15}N-NAM) ≈ 23 s at the same conditions. Note, however, that in protonated basic DM, T_1 at 9.4 T was only 14.5 s. A fundamental difference between 1-^{15}N and ^{15}N-amide is presented by the exposure of the ^{15}N nucleus to its surroundings. This may affect T_1 and hyperpolarization, as observed, and led us to believe that protecting 1-^{15}N may improve the matter. Hence, we tested the DNP of MNAM, where a methyl group protects the 1-^{15}N site.

A sample containing unlabeled MNAM and unlabeled NAM was polarized similarly as described for unlabeled NAM only. After dissolution with neutral DM, the sample was transferred within 17 s and measured at 1 and 9.4 T. Hyperpolarized 1-^{15}N signal of MNAM was observed, revealing T_1 of 274 and 85 s for 1 and 9.4 T, respectively (Fig. 3A, spectrum 6). Again, no 1-^{15}N-NAM signal was found. The experiment was repeated with a pH 9.5 DM, where both 1-^{15}N-MNAM and 1-^{15}N-NAM signals were observed. 1-^{15}N-MNAM T_1 was extraordinarily long once more, with 266 and 67 s at 1 and 9.4 T, respectively. The polarization of 1-^{15}N-MNAM was estimated to be 5 to 10% in both cases (17 s after dissolution). Again, we used DNP parameters optimized for 1-^{15}N-NAM, as no solid-state signal for samples with a natural abundance of ^{15}N was visible.

Chemical transformations of NAM

Only little was reported on observing the enzymatic conversions of the NAM metabolism with NMR in real time. Thus, we set out to observe the actions of NNMT and NAMPT, two enzymes that are, although expensive, commercially available. Although we did not conduct hyperpolarized studies, we made several interesting observations that we would like to share here.

NAM conversion to MNAM, NMN, and NA

Two enzymes involved in the extensive NAM metabolism were available and tested with conventional ^{1}H NMR: NNMT and NAMPT (Figs. 1 and 4). The apparent conversion rate of NAM to MNAM (Fig. 4, A to C) catalyzed by NNMT at 303 K was readily observable with ^{1}H NMR and measured to be k_{NNMT} = (2.36 ± 0.05) × 10^{-5} s^{-1} per mg/ml of enzyme and mM of NAM. The apparent conversion rate of NAM into NMN (Fig. 4, D to F) catalyzed by NAMPT was measured to be k_{NAMPT} = (9.95 ± 0.27) × 10^{-6} s^{-1}

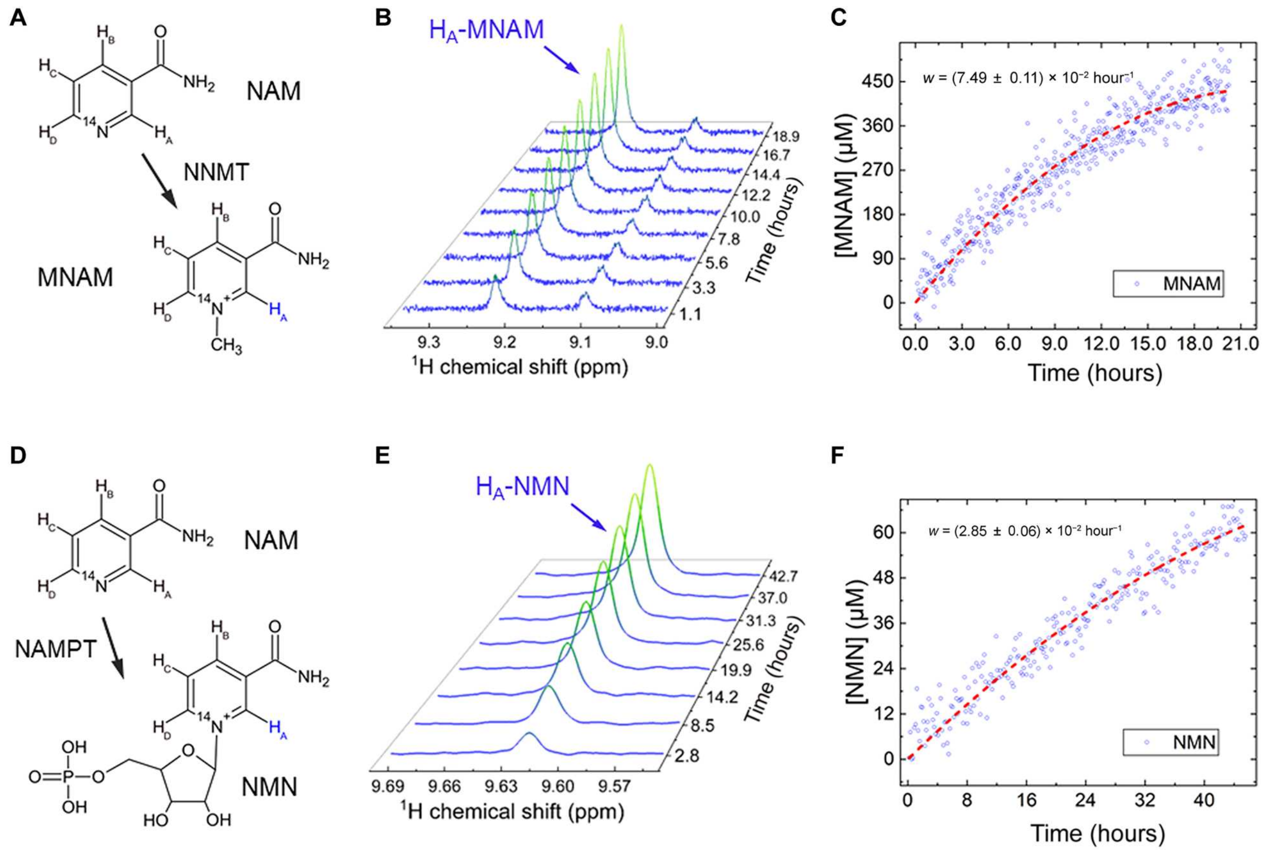

Fig. 4. Chemical transformation of NAM to MNAM and NMN observed with ^{1}H NMR. NNMT-induced conversion of NAM into MNAM (**A** to **C**) was monitored with ^{1}H NMR in situ at 9.4 T over a 21-hour period, revealing a conversion rate of $w = k$[NAM][NNMT] = $(2.30 \pm 0.05) \times 10^{-5}$ s^{-1} NAM (fit—dashed red line, eq. S3) and rate constant of $k_{NNMT} = (2.36 \pm 0.05) \times 10^{-5}$ s^{-1}per mg/ml of enzyme and mM of NAM [TR = 10 s, 800 transients (~2.2 hours) were averaged for each spectrum (B) and 16 transients (~2.7 min) for each data point (C)]. Conversion was estimated to be around 5% with an estimated enzyme degradation of ~21.4% during the experiment. In total, around 171 μg of NNMT (SRP6282) was added to 465 μl of 43.2 mM Trizma buffer with 9.0 mM NAM with 9.0 mM *S*-(5′-adenosyl)-L-methionine chloride (SAM). (**D**) NAMPT-induced conversion of NAM into NMN (**E** and **F**) was monitored with ^{1}H NMR over a 45.5-hour period, revealing a conversion rate of $w = k$[NAM][NAMPT] = $(8.24 \pm 0.23) \times 10^{-6}$ s^{-1} (fit—dashed red line, eq. S3) and a rate constant of $k_{NAMPT} = (9.95 \pm 0.27) \times 10^{-6}$ s^{-1} per mg/ml of enzyme and mM of NAM [TR = 10 s, 2048 transients (~5.7 hours) were averaged for each spectrum (E) and 64 transients (10.7 min) for each data point (F)]. The conversion was estimated to be around 0.7%, with an estimated enzyme degradation of ~44.7% during the experiment. In total, approximately 50 μg of NAMPT (SRP0514) was added to 535 μl of 44.1 mM Trizma buffer with 9.2 mM NAM, 17.7 mM phosphoribosyl pyrophosphate, and 8.7 mM adenosine triphosphate.

per mg/ml of enzyme and mM of NAM at 310 K. These rates were measured using ^{1}H NMR spectroscopy.

During our experiments, we found that NAM converts into NA spontaneously at basic pH, akin to the metabolic step induced by NAHD (Fig. 5). The process seems to be limited only by the amount of accessible hydroxide (OH^-) and the solubility of NA. At pH 13.7 and 335 K, a 0.25 M NAM model solution was converted to NA with a pseudo–first-order rate constant of 1/(33 min). The yield in 1 hour was close to 80% before the experiment was stopped. The reaction rate and NA solubility are higher for higher temperatures (fig. S2).

The spontaneous conversion of NAM to NA allowed us to hyperpolarize ^{15}N-NA without dedicated ^{15}N labeling or synthesis. Before hyperpolarization, we added a small amount of NaOH into the 1-^{15}N-NAM dDNP sample. As a result, NAM was partially converted into NA before hyperpolarization, and both compounds were hyperpolarized. After dissolution with basic DM, both signals were observed at 9.4 T (Fig. 5, E and F) with an average polarization of 5.2%. The chemical shift difference between the two ^{15}N signals was around 1.8 ppm. Note that the chemical transformation continued within the NMR tube due to basic pH. The ongoing conversion is evident in the thermally polarized spectrum, which was averaged during 3 hours after dissolution (Fig. 5F).

Pursuing metabolic transformations of NAM in cells

Despite our awareness that the polarization obtained through the neutral pH protocol for 1-^{15}N NAM DNP was likely insufficient for metabolic studies, we exposed approximately 1.5 million human patient-derived glioma stem-like cells (GSCs) (*55*) and lymphoblast cells (K-562) (*28*) to hyperpolarized 1-^{15}N-NAM. No metabolism of NAM was observed at 9.4-T NMR with ^{15}N hyperpolarized NAM or ^{1}H NMR spectroscopy at thermal equilibrium (cells suspended in 400 μl of cell medium, 200 μl of hyperpolarized and neutralized 1-^{15}N-NAM was added directly before signal acquisition). In comparison, when using the same setup and the K-562 cell line, we observed a rapid conversion of hyperpolarized 1-^{13}C-pyruvate to lactate with a rate of 5.4×10^{-5} s^{-1} (fig. S14).

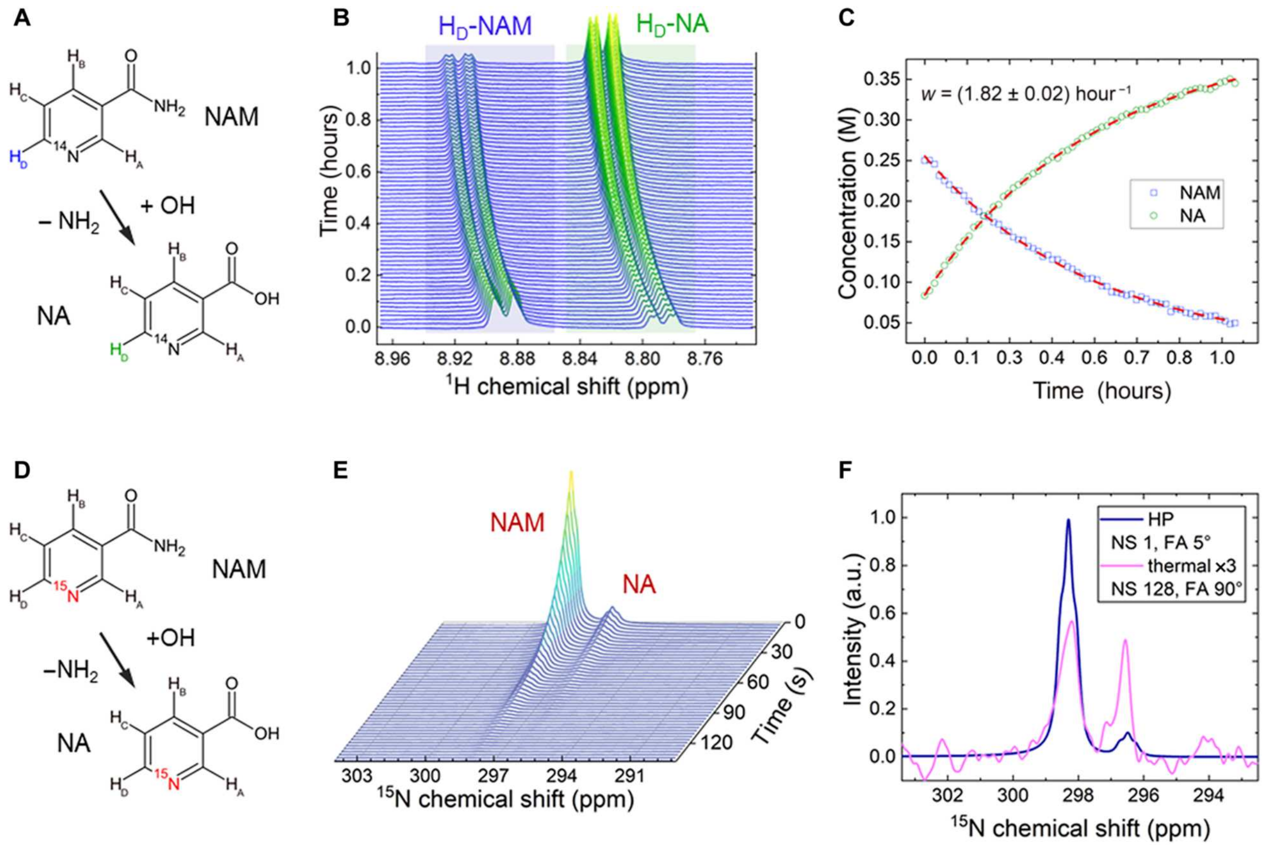

Fig. 5. Chemical transformation of NAM to NA observed at thermal equilibrium and hyperpolarized states. Almost complete conversion of NAM into NA [(**A** and **B**) spectra and (**C**) integrals of H_D signals] was observed for NAM, with an initial concentration of 0.25 M at a pH of 13.7. The kinetics was fit with the exponential decay functions with the shared time *T* of (33.0 ± 0.4) min (red dashed lines). The chemical shift changed due to a decrease in pH throughout the reaction. Adding NaOH to the concentrate before DNP initiated the transformation of NAM to NA (**D**). As a result, both NAM and NA were readily hyperpolarized and yielded T_1 of 23.2 s for 1-^{15}N-NAM and 25.3 s for ^{15}N-NA at 9.4 T and pH of 12.5 (**E**). (**F**) Different signal ratios of NAM/NA at the stage of hyperpolarization (blue) versus at thermal equilibrium after dissolution and following 3 hours of signal averaging (pink) indicate ongoing conversion from NAM to NA. a.u., arbitrary units.

^{15}N MRI of 1-^{15}N-NAM

For ^{15}N-NAM derivatives to be used as a metabolic MRI tracer, we also needed to address imaging aspects. We designed and constructed a single-channel rat-head/mouse-body volume resonator (^{15}N-VOL; Fig. 6, A, B, and D) to check the feasibility of ^{15}N MRI. The performance was compared against a ^{1}H/^{15}N commercial surface coil (^{1}H/^{15}N-SURF, Rapid; Fig. 6C).

^{15}N fast low-angle shot MRI (FLASH MRI) (*5*) of highly concentrated 0.8 M $^{15}NH_4Cl$ acquired in 6.3-hour sequence yielded a signal-to-noise ratio (SNR) of 10 for the surface coil and 3.6 for the volume coil (^{15}N-VOL) (Fig. 6, E and F). ^{15}N FLASH MRIs of hyperpolarized 1-^{15}N-NAM (Fig. 6, G and H) were acquired within 1 s, resulting in an SNR of 14.5 and 4.2 for SURF and VOL coils, respectively. Polarization was quantified to about 5% using the high-resolution 9.4-T NMR.

DISCUSSION

NAM sample and polarization

1-^{15}N-NAM polarization

The 1-^{15}N spin of NAM has already been hyperpolarized with dDNP (*43*) but only at natural abundance. The polarization reported in that work was 1.2%, which is far below the values achieved here (>10%). With these two-digit polarization values, we are approaching the levels needed for in vivo MRI: Today, 20 to 50% of ^{13}C polarization is typically administered for in vivo imaging.

It should be noted that ^{15}N-labeled NAM has been hyperpolarized using another hyperpolarization technique based on parahydrogen (*56*). Here, ^{15}N polarizations of 3.4% (*40*) and ^{15}N images (*41*) were demonstrated. However, it must be noted that these results were achieved in methanol combined with a few mM of iridium-based catalyst—neither are well suited for in vivo applications.

At this time, it appears that DNP is better suited for polarizing NAM because the ^{15}N polarization is higher, the biocompatibility of the solvent and by-products is better, and chemical reactions during the hyperpolarization process are not needed—important factors that have enabled the conduction of clinical studies using DNP.

Further tuning of solid-state 1-^{15}N-NAM polarization

As previously mentioned, the polarization process of ^{15}N with dDNP usually takes longer than for ^{13}C due to its low gyromagnetic ratio. Adding small amounts of [Gd] accelerated the ^{15}N polarization buildup and increased the final solid-state polarization.

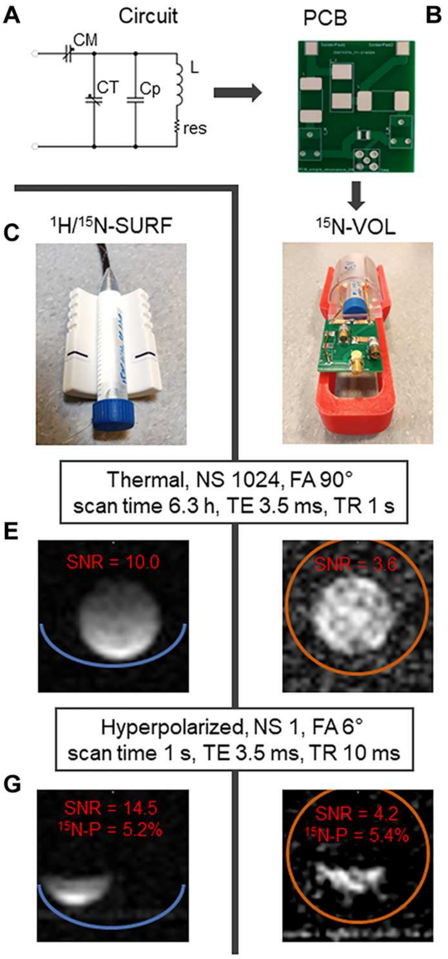

Fig. 6. ^{15}N-MRI probes with phantoms and corresponding thermally and hyperpolarized images. A circuit design (**A**), corresponding printed circuit board (PCB) without circuit elements (**B**), assembled ^{15}N-volume coil (^{15}N-VOL; **D**), and surface (^{1}H/^{15}N-SURF; **C**) are shown. Corresponding thermal (**E** and **F**) and hyperpolarized images (**G** and **H**) demonstrated a 2.8 times higher SNR but much less homogeneous image of ^{1}H/^{15}N-SURF compared to ^{15}N-VOL. ^{15}N-spectroscopy is also possible for 1-^{15}N-NAM (fig. S12). ^{15}N MRI images of 0.8 M $^{15}NH_4$ model solution at thermal equilibrium (E and F) measured with FLASH as follows: TE = 3.5 ms, TR = 1 s, NS = 1024, scan time = 6.3 hours, FOV = 27 mm × 27 mm, matrix size = 32 × 32, slice thickness = 30 mm, FA = 90°, and RF frequency = 17 ppm. Subsequent ^{15}N-MRI of hyperpolarized ^{15}N-NAM (G and H) measured with FLASH as follows: TE = 3.5 ms, TR = 10 ms, NS = 1, nominal FA = 5°, RF frequency = 300 ppm, scan time = 1 s, image size = 32 × 32, slice thickness = 30 mm, and FOV = 32 mm × 32 mm. Right before transferring the hyperpolarized media to the MRI (G and H), 500 μl of the hyperpolarized media was taken to perform quantification of polarization at the high-resolution 9.4-T NMR spectrometer as described previously. Acquisition of NMR spectra for quantification of polarization happened 20 to 30 s after dissolution, as did the acquisition of MRI.

However, 1 mM and above concentrations in the dDNP sample decreased the liquid-state T_1 after dissolution. Considering the successful utilization of cross-polarization to polarize ^{13}C and ^{15}N nuclei of various compounds (*57*, *58*), its potential for further improvement of ^{15}N dDNP of NAM and its derivatives should be explored.

The ratio of solid-state signal decay to signal buildup, T_{decay}/T_{b-up} ≈20, was similar to that of pyruvate (*59*). This finding is unexpected because pyruvate time constants were roughly four to five times shorter (*59*) than for 1-^{15}N-NAM (Fig. 2). It is possible that the radicals clustered in our NAM samples, resulting in a longer polarization buildup time of up to 6 hours because of slower polarization propagation through the sample. However, the glassiness of the samples after freezing in liquid nitrogen was found to be high and consistent. Polarization buildup and final polarization may be slightly accelerated by further refining the sample composition (amount and type of glassing agent or radical). However, due to the slow polarization buildup, this would be a rather long and expensive process. In addition, it does not promise much improvement, and improvement does not appear to be warranted.

The polarization buildup rate for TEMPO was similar to that of both trityls, AH111501 and OX063, although with a lower polarization gain. In the case of TEMPO, polarization is likely inefficient due to the about four times broader resonance compared to trityl (*60*). A radical with an even narrower resonance than trityls AH111501 and OX063 would be more suitable for ^{15}N with its relatively small gyromagnetic ratio (*61*).

Physical and chemical properties of 1-^{15}N-NAM

Effect of pH on ^{15}N resonance

The ^{15}N spectrum offers a very broad chemical shift variation, which is often pH-dependent (*43*). 1-^{15}N-NAM chemical shift changes from around 300 to 200 ppm when pH changes from 2 to 6. Although it is not within the physiological range for many tissues, NAM could be used as a pH-sensing molecule for acidic regions in the body (e.g., kidneys, stomach, or inflamed regions).

Effect of temperature on ^{15}N relaxation

We found that T_1 of ^{15}N-NAM increased steeply with temperature (up to a certain point; fig. S8). T_1 of 1-^{15}N-NAM was doubled by going from room temperature to body temperature. This effect may be attributed to chemical exchange and molecular motion. It will be helpful for in vivo imaging and likely helps to preserve some polarization directly after dissolution with the overheated DM.

Contamination of the dissolution module

The T_1 of 1-^{15}N-NAM was found to be very sensitive, overall. Although intersample variability was low, the long-term differences were noticeable. While we did obtain some clues, we were unable to determine the cause of this. We found that the heating module was corroded after conducting more than a hundred experiments, suspecting that ions could cause the observed relaxation; exchanging this part of the dissolution module, however, did not improve T_1 values substantially. During that time, we also regularly polarized 1-^{13}C-pyruvate, and its ^{13}C T_1 equal to (58 ± 7) s at 9.4 T (n = 14) was not varying like the NAM T_1. Hence, we do not question the reproducibility of the polarization or our procedures and rather suspect more elusive mechanisms.

Hyperpolarization lifetimes achieved with commercial NAM with a natural abundance of ^{15}N and in-house ^{15}N-labeled NAM resulted in similar T_1 values measured at 1 and 9.4 T. This indicates that the purity of 1-^{15}N-NAM was not compromised and was not the source of variability.

It was striking that twice the amount of DMs resulted in a longer liquid-state ^{15}N T_1 (fig. S4). Note that the concentrations of NAM and radical in the liquid state remained the same in some cases. This suggests that the differences in NAM and radical concentrations are not the cause for the variation of T_1. We repeated the experiment with the doubled amount of DM for hyperpolarized 1-^{13}C-pyruvate

and did not observe any effects on ^{13}C T_1. This indicates that these "impurities" (e.g., from the dissolution module or dissolved oxygen) or this effect is tailored to molecules such as 1-^{15}N-NAM. It should be investigated in more detail by studying similarly exposed ^{15}N sites like in NAM.

Fast relaxation of 1-^{15}N at neutral pH and low magnetic fields

Using our on-site pH neutralization procedure, we observed hyperpolarized 1-^{15}N-NAM at 0.57, 1, 7, and 9.4 T under neutral pH conditions. Therefore, we estimated that the lifetime of ^{15}N hyperpolarization at neutral pH and magnetic fields below 0.2 T is about 2.5 s and measured 7 to 12 s at 1 T and 11 to 17 s at 9.4 T. Note that at basic pH, we observed a much longer lifetime of 42.2 s at 1 T. T_1 values of 22 s were measured for natural abundance 1-^{15}N-NAM at 9.4 T (pH 5.9) hyperpolarized with dDNP (*43*) and 20.2 s in methanol hyperpolarized with parahydrogen (*40*).

Such a fast relaxation at neutral and acidic pH at low magnetic fields is likely due to protonation of 1-^{15}N with a subsequent fast exchange of this hydrogen or the association of NAM with quickly relaxing species. At basic pH, no noticeable signal decay was found when the hyperpolarized sample was transported with or without a transfer magnet. This indicates that the mechanism of fast relaxation at low magnetic fields is strongly pH dependent and is prominent already at relatively high fields of hundreds of mT.

In contrast, T_1 of MNAM was much less affected as follows: for pH 7.5, 1-^{15}N T_1 = 85 s (Fig. 3A, spectrum 6), and for pH 9.5, 1-^{15}N T_1 = 67 s (not shown) at 9.4 T. The observed differences in 1-^{15}N polarization, P(pH 7.5) = 6.9% to P(pH 9.5) = 9.1%, are likely caused by the solid-state polarization period, which was longer for the pH 9.5 sample. This matter needs further investigation using ^{15}N-labeled MNAM.

The presence of a quadrupole, a quickly relaxing nucleus (e.g., ^{14}N with spin-1 in ^{13}C-urea) (*47*, *62*), or a quickly exchanging proton in the vicinity of a hyperpolarized nucleus causes relaxation to accelerate at low magnetic fields (*48*). We estimated the effects of the exchanging hydrogen, which is directly coordinated to 1-^{15}N of NAM (fig. S9). However, we were not able to find this exchanging hydrogen using ^{1}H NMR, ^{1}H-^{15}N EXSY, or heteronuclear single-quantum coherence NMR spectroscopy. Therefore, the exchange rate and the amplitude of the mutual spin-spin interaction were not quantified. However, it is possible to estimate the lower boundary for the exchange rate from the fact that only one sharp line of 1-^{15}N-NAM (with ^{1}H decoupling) was observed over the complete pH range from 1 to 14 (*63*, *64*). This is remarkable because two distinct states of NAM with varying distribution exist (protonated H-NAM and NAM). The size of the magnetic field and the ^{15}N chemical shift distance between H-NAM and NAM states indicate a hydrogen exchange constant of 10^8 s^{-1} or more (fig. S9). Such a fast exchange can be the reason for fast relaxation at relatively high magnetic fields (fig. S10). Additional ^{15}N T_1 field-cycling (*65*, *66*) experiments would help to reveal the exact mechanism behind this finding.

In another study, ^{14}N's were substituted with ^{15}N's (*62*); this was found to decrease the speed of relaxation by a factor of around 3 at zero fields. These effects are less visible at higher fields (above 1 T), which supports our theory. We never observed a major difference in ^{15}N lifetime at 9.4 T when pH was neutral or basic, while transfer at low fields and neutral pH was not feasible. It is possible that additional ^{15}N labeling at the amide group of the NAM reduces relaxation such that transfer at neutral pH can be performed. In addition, one should consider the deuteration of the NAM protons, which was shown to be feasible (*67*).

Hyperpolarization of the amide of NAM

The highest signal of hyperpolarized ^{15}N amide of 1-^{15}N enriched and natural abundance NAM was observed using deuterated neutral DM. Likely, the exchange of protons, in this case, is slow, leading to long T_1 of 58 s at 9.4 T and good signal preservation. As ^{15}N amide has such a long T_1 and fast relaxation at neutral pH does not pose a problem, perhaps it is the nucleus of choice of NAM for in vivo analysis. Unfortunately, its chemical shift variability after chemical reactions guided with NAMPT or NNMT is expected to be much lower than for the 1-^{15}N site. For example, the amide resonance from NAM to MNAM only changes by around 1.4 ppm, while the 1-^{15}N shifts by 95 ppm. On the other hand, hyperpolarized ^{15}N-amide will be the most sensitive to the metabolism of NAM-^{15}N-amide (δ_N = 106.4 ppm) to NA and ammonia (δ_N = 23.3 ppm). In the same process, 1-^{15}N only shifts by 1.8 ppm.

Hyperpolarization of NA, MNAM, and NR

These four compounds—NAM, NA, MNAM, and NR—have the highest and close to or exceeding 1 M solubility among all the NAM-related metabolites mentioned (Fig. 1). Therefore, they were considered to be promising for dDNP and subsequent MRI. The additional protection of the 1-N from proton exchange suggests that NR and MNAM hyperpolarized with DNP should not suffer from fast relaxation at low magnetic fields like NAM and could be even more viable for imaging than NA and NAM.

We found a cost-efficient process for synthesizing ^{15}N-labeled NA from NAM. The ^{15}N labeling of NAM has been established already (*68*), but there were no convenient ways of producing ^{15}N-NA. Basic pH with access to NaOH led to conversion from NAM to NA, as observed in an aqueous solution (Fig. 5, A to C) and in the DNP sample (Fig. 5, D to F). As a result, using the same dDNP parameters as for NAM, NA was hyperpolarized. T_1 of NA was found to be similar to that of NAM.

We also attempted to hyperpolarize unlabeled MNAM and NR. MNAM was efficiently co-polarized with naturally abundant ^{15}N NAM and showed more than two times longer T_1 than NAM at basic pH. This trend was observed at 0.57, 1, and 9.4 T, rendering it a feasible alternative to NAM for in vitro and in vivo spectroscopy. The 1-methyl group apparently protects 1-^{15}N and mitigates relaxation effects through the proposed proton exchange mechanism. This effect is most evident at neutral pH, where 1-^{15}N-MNAM was found to preserve 1-^{15}N polarization pH, unlike 1-^{15}N-NAM. 1-^{15}N-MNAM can be synthesized from 1-^{15}N-NAM (*69*–*71*). Synthesis and detailed analysis of 1-^{15}N-MNAM hyperpolarization will be the subject of our future work. The attempt to hyperpolarize naturally abundant NR was not successful, likely due to lower solubility and subsequently low concentration of ^{15}N in the final sample. In addition, the sample composition for DNP could not be optimized due to the absence of a ^{15}N label. Shabalin *et al.* (*72*) reported a large-scale synthesis of NR from labeled 1-^{15}N-NAM. NR is interesting because of its rapid uptake and metabolic conversion to NMN by NAM riboside kinase (NMRK) or to NAM by nucleoside phosphorylase (*72*).

Applications of hyperpolarized NAM

Probing NAMPT

NAMPT converts NAM into NMN (Figs. 1 and 4, D to F). The liver has the highest intracellular NAMPT (iNAMPT) activity of any organ (*73*). Aging, obesity, and chronic inflammation reduce iNAMPT and, consequently, NAD^+. In mammals, an extracellular form of NAMPT (eNAMPT = visfatin = B cell colony–enhancing factor 1 PBEF1) is secreted by adipocytes and possibly hepatocytes and it was shown to be more enzymatically active than iNAMPT (*74*).

Plasma NMN is distributed to tissues and organs and probably modulates glucose-stimulated insulin secretion in pancreatic β cells (*75*). Recent findings suggest that NMN uptake is possible through SLC12A8 transporters (*76*) or via equilibrated nucleoside transporters following cleavage of NMN to NR by CD73 (*77*).

A 2-min exposure of K-562 to NAM at body temperature induced an >50% conversion of the intracellular NAM via NMN to NAD. The rate of biosynthesis was 10^5 molecules per second per cell, mostly renewing the pool of constantly consumed NAD (*28*). In our in vitro studies, we obtained NAMPT-induced conversion of NAM to NMN with the rate of $k_{\mathrm{NAMPT}} = (9.95 \pm 0.41) \times 10^{-7}$ s^{-1} per mg/ml of NAMPT and mM of NAM. Although pH, temperature, and solution were according to the literature recommendations (*78*), conversion appears to be very slow compared to lactate dehydrogenase (LDH) activity (fig. S13). Other work showed that using higher NAM concentrations of 60 mM can lead to 9 mM produced NMN using NAMPT (1 mg/ml) using a thoroughly optimized procedure (*78*). We obtained a concentration of only about 40 μM NMN using NAMPT (around 0.1 mg/ml). Unfortunately, due to the high costs of the pure NAMPT enzyme, we were unable to repeat such experiments with a 10-fold increase in its concentration as described in (*78*). Low enzyme concentration and possible degradation of the enzyme activity are the main reasons for its lower activity in our case.

Probing NNMT

NNMT converts NAM into MNAM (Figs. 1 and 4, A to C). The relevance of NNMT (*36*) for DNA methylation (*79*), immune reaction, and cancer (*80*) in the human body as well as possibly diabetes (*81*) makes it an interesting imaging target. In our in vitro studies, we measured NAM to MNAM by NNMT-induced conversion rate of $k = (1.50 \pm 0.09) \times 10^{-6}\ s^{-1}$ per mg/ml of NNMT and mM of NAM. Our conversion rate for NNMT was more than four times higher compared to that measured for NAMPT.

Isolated enzyme metabolism (NNMT and NAMPT) suggests that such metabolism can take place even without an intact cell membrane or infrastructure. It is likely, however, that the cell microenvironment biases enzyme efficiency in a favorable manner.

Hyperpolarized 1-^{15}N-NAM has the potential to become a tool to better understand organ-specific NAMPT and NNMT activity, as well as NAM uptake and NAD metabolism. We expect that NAM can be used, for example, to study tumor environments or to better comprehend the development of glucose-related diseases such as diabetes mellitus type 2. Both are associated with altered NAM metabolism (*31*, *36*, *79*–*81*).

Cell metabolic studies

Using hyperpolarized 1-^{15}N-NAM added to 1.5 million GSCs or K-562 cells, we did not observe any metabolic transformations. This finding was unexpected as it is well known that GSCs particularly exhibit increased expression of NNMT (*55*) and NAMPT. GSCs are highly relevant for the progression of glioblastomas as highly malignant brain tumors, and especially for the development of recurrences after multimodal therapy (*82*). Hence, monitoring the metabolism and, thus, the activity of specifically these cell types using 1-^{15}N-NAM would be of particular interest. We think that there are at least two reasons why this happened. First, after neutralization, we lost a substantial amount of polarization (down to one-half or one-fifth of the initial polarization, as already discussed). However, neutralization is necessary to examine vital cells in vitro. Another notable fact is that both NNMT and NAMPT exhibit relatively low conversion rates. Hence, the conversion was not fast enough on the time scale of the lifetime of hyperpolarized 1-^{15}N NAM (30 s at 9.4 T).

However, as stated above, using K-562 cells, it was shown (*28*) that following a 2-min exposure to 50 μM NAM, more than 50% of intracellular NAM was converted to NAD via NMN. Therefore, we also tried monitoring NAM to NMN or MNAM conversion in K-562 cells using ^{15}N hyperpolarized or ^{1}H NMR. Again, the sensitivity was not high enough to observe any changes.

NAM metabolism was previously observed in vitro with NMR using metabolic extracts from human embryonic kidney cells (HEK293) (*72*). After an unspecified time of NAM exposure and metabolite extraction, a ^{1}H spectrum was accumulated for around 45 min by using a 16.4-T system with a cryo-cooled probe. Only under these conditions was the SNR feasible for observing this tiny and slow enzymatic reaction with concentrations in the μM range. It is important to bear in mind that the extraction of metabolites may lead to artifacts that do not accurately reflect the conditions inside the cells.

Comparison with the metabolism of pyruvate

Pyruvate is widely used as a hyperpolarization agent not only because it is indicative of the Warburg effect (*83*) but also because its metabolic transformations are very fast. We demonstrated that the complete conversion of 2.12 mM pyruvate to lactate within 2 min can be induced by LDH (0.24 μl/ml) (fig. S13). Such fast activity is about 2×10^5 times faster than the conversion rates of NAMPT and NNMT measured here. Therefore, ^{1}H NMR or hyperpolarized ^{13}C NMR and MRI is successful in following the conversion of pyruvate to lactate (*84*). Using 1.5 million K-562 cells, we observed a relatively fast conversion rate of hyperpolarized ^{13}C-pyruvate to lactate with $w = 5.3 \times 10^{-5}\ s^{-1}$ (fig. S14).

This suggests that cell vitality or experiment procedure was not the limiting factor when we tried to observe NAM metabolism. To study the enzymatic activity of NAM, we propose extracting proteins and enzymes from cells. This produces samples with a higher enzyme concentration and thus enabling faster conversion. In addition, following the extraction, the uptake of NAM through the cell membranes will not cause any delays as the chemical conversion will take place immediately within the solution.

Subsecond ^{15}N MRI of NAM

Using hyperpolarized 1-^{15}N-NAM, the imaging at 7 T was possible with a sufficient SNR ranging from 5 to 13 (Fig. 6). This was achieved by using the FLASH sequence with a 5° excitation angle for each line in *k*-space with a rapid acquisition time of 1 s. Acceleration techniques as well as the use of the balanced steady-state free precession (BSSFP) sequence and variants (*85*) will further improve the preservation of hyperpolarization and image quality.

The ^{15}N-VOL coil was designed such that it can be built in 1 day and at the cost of only about 500 euros. This "high" cost derives

mostly from two high-power, nonmagnetic variable capacitors, while the three-dimensional (3D)–printed parts and circuit board cost below 10 euros. The SNR averaged across the axial slice for the ^{15}N-VOL coil was about 3 times lower compared to the commercial ^{1}H/^{15}N-surface coil (Fig. 6), which costs about 30 times more. In addition, to achieve the same excitation angle, the duration of the pulse was about three times longer for the ^{15}N-VOL coil with the same radio frequency (RF) power. Lower SNR is an effect of a lower filling factor and a higher average distance of the sample to the coil. However, the volume design also results in a homogeneous excitation and image intensity across the whole volume, making the ^{15}N-VOL coil more appealing for use together with accelerated techniques such as BSSFP.

Implications of the hyperpolarizing NAM and its derivatives in biocompatible solutions

In vivo metabolic imaging using hyperpolarized tracers has the potential to become a versatile diagnostic tool. However, up to now, only a few ^{13}C-labeled metabolites have been successfully used in vivo: some sugars (*86*), pyruvate (*16*), fumarate (*87*), and short fatty acids (*88*). Other molecules, such as amino acids (*89*, *90*), were not strongly polarized in otherwise identical conditions.

Independent studies of clinically approved antibiotic $^{15}N_3$-metronidazole hyperpolarized with dDNP revealed a ^{15}N polarization over 6% with an impressive relaxation time of up to 343 s at 4.7 T (*91*). Such long relaxation times enabled rat-head ^{15}N spectroscopy. This study proves an interest to ^{15}N tracers as an alternative to commonly used hyperpolarized ^{13}C tracers. While metronidazole showcases its antibiotic applicability, our work on NAM and its derivatives demonstrate their potential as physiologically viable metabolites with broad diagnostic capabilities.

Through our extensive studies, we aimed to bring ^{15}N hyperpolarized metabolic tracers (^{15}N-labeled NAM and NA and natural abundance MNAM) to general attention. They probe the metabolism of NAM to NAD and other metabolites. The drastic variation in ^{15}N chemical shifts during metabolization of NAM combined with a long relaxation time of 30 to 50 s for NAM or 260 s for MNAM depending on field strength makes ^{15}N-spectroscopy very sensitive for NAM-related metabolic pathways.

We found various effects from pH, temperature, magnetic field, and sample compositions on T_1 and polarization, which were not previously reported. Each parameter can drastically change the polarization to the point of being completely unobservable. One of the main challenges we experienced in our work is that 1-^{15}N-NAM cannot be neutralized directly using a buffered neutral DM. This is because the polarization is entirely lost during transportation from the polarizer to the MRI or NMR. Therefore, we proposed performing neutralization quickly, right next to the measuring site or inside of the MRI bore, which partially preserved polarization. Our explanation for this effect is the rapid exchange of proton at the 1-^{15}N site. While the methyl group in MNAM prohibits this effect, an additional relaxation study as a function of magnetic fields will help to refine the relaxation mechanism strategy for conserving the 1-^{15}N-NAM polarization. Nevertheless, we are already reliably achieving ^{15}N polarization at a rate of around 13%, which was found to be sufficient for rapid ^{15}N MRI using commercial or low-cost in-house constructed coils.

MATERIALS AND METHODS

Chemicals

Sample preparation

Samples for dDNP were prepared by mixing NAM (72340, CAS: 98-92-0, Sigma-Aldrich) or in-house synthesized 1-^{15}N-NAM with trityl radical (AH111501 or OX063, Polarize) or nitroxyl radical (TEMPO, 426369, CAS: 2564-83-2, Sigma-Aldrich) in deionized water with glycerol (G9012, CAS: 56-81-5, Sigma-Aldrich) or trehalose (90210, CAS: 6138-23-4, Sigma-Aldrich) (*44*, *45*). In some cases, a Gd contrast agent was added to increase the rate and amplitude of the polarization buildup (*50*) ([Gd], gadobutrol Gd-DO3A-butrol, 1 mmol/ml; Gadovist, Bayer). If needed, NaOH was used to keep pH > 12 during the process and to induce NAM to NA conversion before DNP.

A typical sample consisted of 130 mg of water and 50 mg of glycerol or 70 mg of trehalose, 100 mg of 1-^{15}N-NAM, and 10 mg of trityl radical, resulting in a sample volume of 250 μl. The final concentrations of trityl radical and 1-^{15}N-NAM were around 28 mM and 3.4 M, respectively. When prepared, the solution was stored at −24°C. Before use, the vial was warmed up (in hands) and vortexed for 5 min. The typical sample size was 50 mg. Different sample compositions are noted, and a complete list of all samples is provided in the Supplementary Materials.

The glassiness was checked visually by placing a 3-μl droplet into the liquid nitrogen. Such a sample was considered glassy if a transparent drop without visible defects was achieved.

^{15}N-nicotinamide

1-^{15}N-NAM was synthesized in a two-step reaction from NAM (72340, CAS: 98-92-0, Sigma-Aldrich) via the Zincke salt followed by nitrogen exchange with $^{15}NH_4Cl$ (299251, CAS: 39466-62-1, Sigma-Aldrich). In the first step, the Zincke salt of NAM was formed with 1-chloro-2,4-dinitrobenzene (237329, CAS: 97-00-7, Sigma-Aldrich) in DMSO (*40*). The resulting compound was a slightly yellow powder, which was subsequently reacted with $^{15}NH_4Cl$ to obtain 1-^{15}N-NAM as a white powder. In some cases, there was a yellow tint from the presence of 2,4-dinitroaniline after the chromatographic workup, which could not be separated chromatographically. In these cases, we propose developing an additional purification step with activated charcoal before purification via column chromatography or additional recrystallization in ethyl acetate after the column chromatography. The best yield was 40% for ^{15}N-NAM with 91 ± 2% ^{15}N enrichment. The synthesis is detailed in the Supplementary Materials.

Neutral DM

The DM with a pH of 7 was prepared by mixing 300 mg of Trizma preset crystals (pH 7.6, average M = 149.0 g/mol, T7943, Sigma-Aldrich), 10 mg of EDTA (11280, CAS: 9002-07-7, SERVA), 300 mg of NaCl (S9888, CAS: 7647-14-5, Honeywell), and 50 mg of NaOH (1355, CAS: 1310-73-2, ChemSolute) in 50 ml of deionized water or 99.9% D_2O (151882, Sigma-Aldrich) and stored at room temperature.

Basic DM

The DM with a pH of 13 was prepared by mixing 300 mg of Trizma preset crystals, 10 mg of EDTA, and 300 g of NaOH in 50 ml of deionized water or 99.9% D_2O and stored at room temperature.

Acidic DM

The DM with a pH of 0.5 was prepared by mixing 300 mg of Trizma preset crystals, 10 mg of EDTA, and 0.5 ml of concentrated HCl in

50 ml of deionized water or 99.9% D_2O and stored at room temperature.

Hyperpolarization and NMR signal acquisition
Dissolution dynamic nuclear polarization
All dDNP experiments were performed using a cryogen-free dDNP system (SpinAligner, Polarize) (*59*, *92*) at ~1.4 K and 6.7 T. An MW frequency between 187.07 and 187.19 GHz with 10- to 45-mW power was used for polarization. The optimal MW frequency was calibrated for each sample batch by varying the MW frequency (*59*). For each DNP experiment, the indicated amount of the concentrate (typically 50 mg) was taken from the stock, filled into the sample cup, and lowered into the MW cavity at ≈1.3 K. DNP was initiated by continuous wave irradiation at the optimized frequency and power. The buildup of the ^{15}N polarization in the solid state was monitored every 5 to 15 min with a ^{15}N RF pulse of 3°. The flipping angle (FA) of the in-built NMR was calibrated for ^{15}N before the experiments. After dissolution with 4 to 8 ml of superheated (~200°C, 11 bar) DM (acidic DM: pH 1.5, neutral DM: pH 7, basic DM: pH 13), the sample was transferred to an NMR system and detected 17 to 30 s later.

NMR and MRI
^{15}N MR signals were acquired using a 1-T ^{15}N benchtop NMR (Spinsolve Nitrogen, Magritek), a 9.4-T wide bore NMR (WB400, Avance NEO, Bruker) with a 5-mm BBFO probe, a 7-T MRI (BioSpec 70/30, Bruker), and a benchtop, 0.57-T, 10-mm MRI system ("magspec" magnet unit, "drive L" console unit, Pure Devices). Two MRI coil settings for 7-T MRI were compared (figs. S11 and S12): a ^{1}H-^{15}N, fixed-tune, linear surface coil (^{1}H/^{15}N-SURF, 30 mm, O-XL-HL-070, Rapid Biomedical) or an in-house–built, 3D-printed, linear ^{15}N saddle-shaped volume coil (^{15}N-VOL, length = 52 mm, inside diameter = 46 mm), the latter in combination with a ^{1}H quadrature resonator (112/086 QSN, Bruker). The ^{1}H/^{15}N-SURF was placed directly under the vial to maximize its sensitivity.

For ^{15}N-VOL, a single-wire saddle-shaped coil was wound around the 3D-printed housing and animal bed. The resulting single-nucleus linear volume coil was tuned to a ^{15}N frequency at 7 T. The coil assembly was connected to the moving table. We used a commercial volume ^{1}H resonator for localization, shimming, and frequency adjustment. ^{15}N-VOL was designed such that it can easily be moved into the ^{1}H volume resonator with a phantom or animal.

The ^{15}N images at thermal equilibrium were acquired using the following parameters of the FLASH sequence: echo time (TE) = 3.5 ms, repetition time (TR) of 1 s, number of averages (NS) of 1024, nominal FA of 90°; RF frequency was set in resonance with $^{15}NH_4Cl$ at 17 ppm, scan time of 6.3 hours, matrix size of 32 × 32, slice thickness of 30 mm, and field of view (FOV) of 27 mm × 27 mm. To increase the relaxation rate, we added ~10 mM [Gd] such that TR of 1 s was longer than 5 T_1.

The ^{15}N hyperpolarized images were acquired using the following parameters of the FLASH sequence: TE = 3.5 ms, TR = 10 ms, NS = 1, nominal FA = 5°, RF frequency in resonance with 1-^{15}N-NAM at 300 ppm, scan time of 1 s, matrix size of 32 × 32, slice thickness of 30 mm, and FOV = 32 mm × 32 mm.

Sample transfer
For transportation to 1- and 9.4-T measuring sites, a 0.5- to 1-T Halbach magnet array was used as described by Capozzi *et al.* (*93*). However, no measurable effect on liquid-state polarization was observed whether or not the magnet was used. Because of the short proximity to the 1- and 7-T MR systems, no transfer magnet was used.

Quantification
Quantification methods
NMR spectra were quantified by manual integration after manual phase correction, line broadening, and baseline correction (MestReNova 14.2.2, Mestrelab Research S.L.). MRI images were analyzed using the manufacturer′s software (Paravision 360, Bruker).

Signal enhancement
The signal enhancement ε was quantified concerning the accumulated signal of the same sample in thermal equilibrium using (Eq. 1)

$$P = P_{\text{therm}} \cdot \varepsilon = \frac{S^{\text{HP}}}{S^{\text{TP}}} \cdot \frac{\text{NS}_{\text{acq}}^{\text{TP}}}{\text{NS}_{\text{acq}}^{\text{HP}}} \cdot \frac{\sin(\alpha^{\text{TP}})}{\sin(\alpha^{\text{HP}})} \cdot \frac{\text{RG}^{\text{TP}}}{\text{RG}^{\text{HP}}} \quad (1)$$

where P_{therm} is the polarization in thermal equilibrium, S^{x} is the integral of the respective signal, $\text{NS}_{\text{acq}}^{x}$ is the number of accumulated spectra, α^{x} is the excitation angle, and RG^{x} is linear receiver gain for hyperpolarized (x = HP) and thermally polarized (x = TP) NMR spectra.

Thermally polarized liquid-state NMR
Thermally polarized liquid-state NMR spectra were acquired with high-resolution NMR at 9.4 T, ^{1}H decoupling, and the following acquisition parameters: number of accumulations $\text{NS}_{\text{acq}}^{\text{TP}}$ = 64, flip angle α^{TP} = 90°, and TR = 170 s. A typical SNR of 20 was obtained. ^{15}N signal intensities were quantified using these quantification methods. The selected integration region around the hyperpolarized signal was ±3 ppm. The polarization was quantified only at 7 and 9.4 T. The SNR for the thermal ^{15}N signal was below 2.8 at 1 T after 30,000 acquisitions with TR = 2 s ([Gd] doped sample) and thus not practical and sensitive enough.

Liquid-state polarization decay
The decaying hyperpolarization was sampled with a α^{HP} = 5° pulse every 3 to 12 s. To quantify the lifetime of hyperpolarization, T_1^{HP}, a monoexponential decay function was fit to the data, yielding the apparent/observed constant T_1^{obs} (Eq. 2)

$$S^{\text{obs}}(t) = S_0 \cdot e^{-\frac{t}{T_1^{\text{obs}}}} \quad (2)$$

To obtain the longitudinal relaxation time T_1, T_1^{obs} was corrected in all cases if not otherwise stated, considering the polarization consumed by the repetitive RF excitations with angle α^{HP} as (*59*)

$$T_1 = T_1^{\text{obs}} \left\{ 1 + \frac{T_1^{\text{obs}}}{TR} \ln[\cos(\alpha^{\text{HP}})] \right\}^{-1} \quad (3)$$

Biological materials and ethical requirements
Human patient-derived GSCs were generated by dissociation of tumor material obtained by surgical dissection at the Department of Neurosurgery (Kiel, Germany) with approval of the ethics committee of the University of Kiel, Germany, after written informed consent of donors (file reference: D471/15 and D524/17) and following the Helsinki Declaration of 1975 as described previously (*94*). Cells were cultured under stem-like cell conditions in F12 medium supplemented with B27 supplement (Thermo Fisher Scientific, Waltham, MA, USA), 2 mM L-glutamine, and 1% penicillin-

streptomycin (10,000 U/ml). The epidermal growth factor (EGF) and basic fibroblast growth factor (bFGF) were added at a concentration of 10 ng/ml. GSCs were characterized by the formation of neurospheres, the ability to survive and proliferate under stem cell conditions, and to differentiate into more mature cells, which was proven as described previously (*94*–*96*). The purity of the GSCs was ascertained by immunostaining with cell type–specific markers and by the absence of contamination with mycoplasms. For measurements, 1.5×10^6 cells were resuspended in 200 μl of medium (F12/B27 + 10 ng/ml bFGF + EGF; pH 7.09 when kept in 5% CO_2, sodium bicarbonate buffer system), transferred into the NMR tube, and were ready for further processing.

The human chronic myelogenous leukemia cell line K-562 was cultured in customized NAM-free Dulbecco's modified Eagle's medium (DMEM) (Gibco) with 10% dialyzed fetal bovine serum (Sigma-Aldrich, F0392) at 37°C and 5% CO_2 with 95% humidity. For measurements, 2×10^6 cells were resuspended in 400 μl of culture medium.

Supplementary Materials

This PDF file includes:
Sections S1 to S18
Figs. S1 to S15
Tables S1 and S2

REFERENCES AND NOTES

1. S. M. Grundy, Pre-diabetes, metabolic syndrome, and cardiovascular risk. *J. Am. Coll. Cardiol.* **59**, 635–643 (2012).
2. P. S. Kishnani, R. R. Howell, Pompe disease in infants and children. *J. Pediatr.* **144**, S35–S43 (2004).
3. M. V. Liberti, J. W. Locasale, The Warburg effect: How does it benefit cancer cells? *Trends Biochem. Sci.* **41**, 211–218 (2016).
4. R. J. DeBerardinis, N. S. Chandel, Fundamentals of cancer metabolism. *Sci. Adv.* **2**, e1600200 (2016).
5. A. Haase, J. Frahm, D. Matthaei, W. Hänicke, K.-D. Merboldt, FLASH imaging: Rapid NMR imaging using low flip-angle pulses. *J. Magn. Reson.* **213**, 533–541 (1986).
6. B. Fidock, N. Barker, N. Balasubramanian, G. Archer, G. Fent, A. Al-Mohammad, J. Richardson, L. O'Toole, N. Briffa, A. Rothman, R. van der Geest, R. Hose, J. M. Wild, A. J. Swift, P. Garg, A systematic review of 4D-flow MRI derived mitral regurgitation quantification methods. *Front. Cardiovasc. Med.* **6**, 103 (2019).
7. M. Durst, U. Koellisch, A. Frank, G. Rancan, C. V. Gringeri, V. Karas, F. Wiesinger, M. I. Menzel, M. Schwaiger, A. Haase, R. F. Schulte, Comparison of acquisition schemes for hyperpolarised ^{13}C imaging. *NMR Biomed.* **28**, 715–725 (2015).
8. G. Muehllehner, J. S. Karp, Positron emission tomography. *Phys. Med. Biol.* **51**, R117–R137 (2006).
9. G. Mariani, L. Bruselli, T. Kuwert, E. E. Kim, A. Flotats, O. Israel, M. Dondi, N. Watanabe, A review on the clinical uses of SPECT/CT. *Eur. J. Nucl. Med. Mol. Imaging* **37**, 1959–1985 (2010).
10. C. A. Combs, H. Shroff, Fluorescence microscopy: A concise guide to current imaging methods. *Curr. Protoc. Neurosci.* **79**, 2.1.1–2.1.25 (2017).
11. A. N. Pravdivtsev, A. V. Yurkovskaya, R. Kaptein, K. Miesel, H.-M. Vieth, K. L. Ivanov, Exploiting level anti-crossings for efficient and selective transfer of hyperpolarization in coupled nuclear spin systems. *Phys. Chem. Chem. Phys.* **15**, 14660–14669 (2013).
12. R. A. Green, R. W. Adams, S. B. Duckett, R. E. Mewis, D. C. Williamson, G. G. R. Green, The theory and practice of hyperpolarization in magnetic resonance using parahydrogen. *Prog. Nucl. Magn. Reson. Spectrosc.* **67**, 1–48 (2012).
13. J. H. Ardenkjær-Larsen, B. Fridlund, A. Gram, G. Hansson, L. Hansson, M. H. Lerche, R. Servin, M. Thaning, K. Golman, Increase in signal-to-noise ratio of >10,000 times in liquid-state NMR. *Proc. Natl. Acad. Sci. U.S.A.* **100**, 10158–10163 (2003).
14. J. R. Birchall, P. Nikolaou, A. M. Coffey, B. E. Kidd, M. Murphy, M. Molway, L. B. Bales, B. M. Goodson, R. K. Irwin, M. J. Barlow, E. Y. Chekmenev, Batch-mode clinical-scale optical hyperpolarization of Xenon-129 using an aluminum jacket with rapid temperature ramping. *Anal. Chem.* **92**, 4309–4316 (2020).
15. P. Bhattacharya, K. Harris, A. P. Lin, M. Mansson, V. A. Norton, W. H. Perman, D. P. Weitekamp, B. D. Ross, Ultra-fast three dimensional imaging of hyperpolarized ^{13}C in vivo. *Magn. Reson. Mater. Phys.* **18**, 245–256 (2005).
16. S. J. Nelson, J. Kurhanewicz, D. B. Vigneron, P. E. Z. Larson, A. L. Harzstark, M. Ferrone, M. van Criekinge, J. W. Chang, R. Bok, I. Park, G. Reed, L. Carvajal, E. J. Small, P. Munster, V. K. Weinberg, J. H. Ardenkjaer-Larsen, A. P. Chen, R. E. Hurd, L.-I. Odegardstuen, F. J. Robb, J. Tropp, J. A. Murray, Metabolic imaging of patients with prostate cancer using hyperpolarized [1-^{13}C]pyruvate. *Sci. Transl. Med.* **5**, 198ra108 (2013).
17. C. H. Cunningham, J. Y. C. Lau, A. P. Chen, B. J. Geraghty, W. J. Perks, I. Roifman, G. A. Wright, K. A. Connelly, Hyperpolarized ^{13}C metabolic MRI of the human heart. *Circ. Res.* **119**, 1177–1182 (2016).
18. R. Aggarwal, D. B. Vigneron, J. Kurhanewicz, Hyperpolarized 1-[^{13}C]-pyruvate magnetic resonance imaging detects an early metabolic response to androgen ablation therapy in prostate cancer. *Eur. Urol.* **72**, 1028–1029 (2017).
19. J. T. Grist, M. A. McLean, F. Riemer, R. F. Schulte, S. S. Deen, F. Zaccagna, R. Woitek, C. J. Daniels, J. D. Kaggie, T. Matys, I. Patterson, R. Slough, A. B. Gill, A. Chhabra, R. Eichenberger, M.-C. Laurent, A. Comment, J. H. Gillard, A. J. Coles, D. J. Tyler, I. Wilkinson, B. Basu, D. J. Lomas, M. J. Graves, K. M. Brindle, F. A. Gallagher, Quantifying normal human brain metabolism using hyperpolarized [1–^{13}C]pyruvate and magnetic resonance imaging. *Neuroimage* **189**, 171–179 (2019).
20. H. Stødkilde-Jørgensen, C. Laustsen, E. S. S. Hansen, R. Schulte, J. H. Ardenkjaer-Larsen, A. Comment, J. Frøkiær, S. Ringgaard, L. B. Bertelsen, M. Ladekarl, B. Weber, Pilot study experiences with hyperpolarized [1-^{13}C]pyruvate MRI in pancreatic cancer patients. *J. Magn. Reson. Imag.* **51**, 961–963 (2020).
21. K. M. Brindle, S. E. Bohndiek, F. A. Gallagher, M. I. Kettunen, Tumor imaging using hyperpolarized ^{13}C magnetic resonance spectroscopy. *Magn. Reson. Med.* **66**, 505–519 (2011).
22. E. Cavallari, C. Carrera, M. Sorge, G. Bonne, A. Muchir, S. Aime, F. Reineri, The ^{13}C hyperpolarized pyruvate generated by ParaHydrogen detects the response of the heart to altered metabolism in real time. *Sci. Rep.* **8**, 8366 (2018).
23. Y. Ding, S. Korchak, S. Mamone, A. P. Jagtap, G. Stevanato, S. Sternkopf, D. Moll, H. Schroeder, S. Becker, A. Fischer, E. Gerhardt, T. F. Outeiro, F. Opazo, C. Griesinger, S. Glöggler, Rapidly signal-enhanced metabolites for atomic scale monitoring of living cells with magnetic resonance. *Chem. Methods* **2**, e202200023 (2022).
24. T. Theis, G. X. Ortiz Jr., A. W. J. Logan, K. E. Claytor, Y. Feng, W. P. Huhn, V. Blum, S. J. Malcolmson, E. Y. Chekmenev, Q. Wang, W. S. Warren, Direct and cost-efficient hyperpolarization of long-lived nuclear spin states on universal $^{15}N_2$-diazirine molecular tags. *Sci. Adv.* **2**, e1501438 (2016).
25. C. Cudalbu, A. Comment, F. Kurdzesau, R. B. van Heeswijk, K. Uffmann, S. Jannin, V. Denisov, D. Kirik, R. Gruetter, Feasibility of in vivo15N MRS detection of hyperpolarized 15N labeled choline in rats. *Phys. Chem. Chem. Phys.* **12**, 5818–5823 (2010).
26. H. Nonaka, R. Hata, T. Doura, T. Nishihara, K. Kumagai, M. Akakabe, M. Tsuda, K. Ichikawa, S. Sando, A platform for designing hyperpolarized magnetic resonance chemical probes. *Nat. Commun.* **4**, 2411 (2013).
27. P. J. Rayner, M. Fekete, C. A. Gater, F. Ahwal, N. Turner, A. J. Kennerley, S. B. Duckett, Real-time high-sensitivity reaction monitoring of important nitrogen-cycle synthons by ^{15}N hyperpolarized nuclear magnetic resonance. *J. Am. Chem. Soc.* **144**, 8756–8769 (2022).
28. A. Olsson, T. Olofsson, R. W. Pero, Specific binding and uptake of extracellular nicotinamide in human leukemic k-562 cells. *Biochem. Pharmacol.* **45**, 1191–1200 (1993).
29. I. V. Linnik, P. J. Rayner, R. A. Stow, S. B. Duckett, G. M. T. Cheetham, Pharmacokinetics of the SABRE agent 4,6-d2-nicotinamide and also nicotinamide in rats following oral and intravenous administration. *Eur. J. Pharm. Sci.* **135**, 32–37 (2019).
30. J. R. Revollo, A. A. Grimm, S. Imai, The NAD biosynthesis pathway mediated by nicotinamide phosphoribosyltransferase regulates Sir2 activity in mammalian cells*. *J. Biol. Chem.* **279**, 50754–50763 (2004).
31. K. Yaku, K. Okabe, K. Hikosaka, T. Nakagawa, NAD metabolism in cancer therapeutics. *Front. Oncol.* **8**, 622 (2018).
32. A. A. Pramono, G. M. Rather, H. Herman, K. Lestari, J. R. Bertino, NAD- and NADPH-contributing enzymes as therapeutic targets in cancer: An overview. *Biomolecules.* **10**, 358 (2020).
33. A. Rongvaux, R. J. Shea, M. H. Mulks, D. Gigot, J. Urbain, O. Leo, F. Andris, Pre-B-cell colony-enhancing factor, whose expression is up-regulated in activated lymphocytes, is a nicotinamide phosphoribosyltransferase, a cytosolic enzyme involved in NAD biosynthesis. *Eur. J. Immunol.* **32**, 3225–3234 (2002).
34. I. Shin-ichiro, Nicotinamide phosphoribosyltransferase (Nampt): A link between NAD biology, metabolism, and diseases. *Curr. Pharm. Des.* **15**, 20–28 (2008).
35. S. Aksoy, C. L. Szumlanski, R. M. Weinshilboum, Human liver nicotinamide *N*-methyltransferase. cDNA cloning, expression, and biochemical characterization. *J. Biol. Chem.* **269**, 14835–14840 (1994).

36. P. Pissios, Nicotinamide *N*-methyltransferase: More than a vitamin B3 clearance enzyme. *Trends Endocrinol. Metab.* **28**, 340–353 (2017).
37. K. N. Murray, J. G. Watson, S. Chaykin, Catalysis of the direct transfer of oxygen from nicotinamide *N*-oxide to xanthine by xanthine oxidase. *J. Biol. Chem.* **241**, 4798–4801 (1966).
38. M. R. L. Stratford, M. F. Dennis, High-performance liquid chromatographic determination of nicotinamide and its metabolites in human and murine plasma and urine. *J. Chromatogr. B Biomed. Appl.* **582**, 145–151 (1992).
39. P. Rovedo, S. Knecht, T. Bäumlisberger, A. L. Cremer, S. B. Duckett, R. E. Mewis, G. G. R. Green, M. Burns, P. J. Rayner, D. Leibfritz, J. G. Korvink, J. Hennig, G. Pütz, D. von Elverfeldt, J.-B. Hövener, Molecular MRI in the Earth's magnetic field using continuous hyperpolarization of a biomolecule in water. *J. Phys. Chem. B* **120**, 5670–5677 (2016).
40. R. V. Shchepin, D. A. Barskiy, D. M. Mikhaylov, E. Y. Chekmenev, Efficient synthesis of nicotinamide-1-^{15}N for ultrafast NMR hyperpolarization using parahydrogen. *Bioconjug. Chem.* **27**, 878–882 (2016).
41. A. Svyatova, I. V. Skovpin, N. V. Chukanov, K. V. Kovtunov, E. Y. Chekmenev, A. N. Pravdivtsev, J.-B. Hövener, I. V. Koptyug, ^{15}N MRI of SLIC-SABRE hyperpolarized ^{15}N-labelled pyridine and nicotinamide. *Chem. A Eur. J.* **25**, 8465–8470 (2019).
42. W. Iali, A. M. Olaru, G. G. R. Green, S. B. Duckett, Achieving high levels of NMR-hyperpolarization in aqueous media with minimal catalyst contamination using SABRE. *Chem. A Eur. J.* **23**, 10491–10495 (2017).
43. W. Jiang, L. Lumata, W. Chen, S. Zhang, Z. Kovacs, A. D. Sherry, C. Khemtong, Hyperpolarized ^{15}N-pyridine derivatives as pH-sensitive MRI agents. *Sci. Rep.* **5**, 9104 (2015).
44. J. R. Brender, S. Kishimoto, G. R. Eaton, S. S. Eaton, Y. Saida, J. Mitchell, M. C. Krishna, Trehalose as an alternative to glycerol as a glassing agent for in vivo DNP MRI. *Magn. Reson. Med.* **85**, 42–48 (2021).
45. M. Kaushik, H. Lingua, G. Stevanato, M. Elokova, M. Lelli, A. Lesage, O. Ouari, Trehalose matrices for high temperature dynamic nuclear polarization enhanced solid state NMR. *Phys. Chem. Chem. Phys.* **24**, 12167–12175 (2022).
46. F. Jähnig, G. Kwiatkowski, A. Däpp, A. Hunkeler, B. H. Meier, S. Kozerke, M. Ernst, Dissolution DNP using trityl radicals at 7 T field. *Phys. Chem. Chem. Phys.* **19**, 19196–19204 (2017).
47. G. D. Reed, C. von Morze, R. Bok, B. L. Koelsch, M. Van Criekinge, K. J. Smith, H. Shang, P. E. Z. Larson, J. Kurhanewicz, D. B. Vigneron, High resolution ^{13}C MRI with hyperpolarized urea: In vivo T_2 mapping and ^{15}N labeling effects. *IEEE Trans. Med. Imaging* **33**, 362–371 (2014).
48. E. Chiavazza, E. Kubala, C. V. Gringeri, S. Düwel, M. Durst, R. F. Schulte, M. I. Menzel, Earth's magnetic field enabled scalar coupling relaxation of 13C nuclei bound to fast-relaxing quadrupolar ^{14}N in amide groups. *J. Magn. Reson.* **227**, 35–38 (2013).
49. M. Durst, E. Chiavazza, A. Haase, S. Aime, M. Schwaiger, R. F. Schulte, α-Trideuteromethyl[^{15}N]glutamine: A long-lived hyperpolarized perfusion marker. *Magn. Reson. Med.* **76**, 1900–1904 (2016).
50. A. Capozzi, S. Patel, W. T. Wenckebach, M. Karlsson, M. H. Lerche, J. H. Ardenkjær-Larsen, Gadolinium effect at high-magnetic-field DNP: 70% 13C polarization of [U-^{13}C] glucose using trityl. *J. Phys. Chem. Lett.* **10**, 3420–3425 (2019).
51. J. Kowalewski, L. Mäler, *Nuclear Spin Relaxation in Liquids: Theory, Experiments, and Applications* (Taylor & Francis, 2006).
52. M. Krajewski, P. Wespi, J. Busch, L. Wissmann, G. Kwiatkowski, J. Steinhauser, M. Batel, M. Ernst, S. Kozerke, A multisample dissolution dynamic nuclear polarization system for serial injections in small animals. *Magn. Reson. Med.* **77**, 904–910 (2017).
53. H. Gutte, A. E. Hansen, M. M. E. Larsen, S. Rahbek, S. T. Henriksen, H. H. Johannesen, J. Ardenkjaer-Larsen, A. T. Kristensen, L. Højgaard, A. Kjær, Simultaneous hyperpolarized ^{13}C-Pyruvate MRI and ^{18}F-FDG PET (HyperPET) in 10 dogs with cancer. *J. Nuc. Med.* **56**, 1786–1792 (2015).
54. D. D. Perrin, *Dissociation Constants of Organic Bases in Aqueous Solution* (Butterworths, 1972), vol. 1 of *International Union of Pure and Applied Chemistry. Commission on Electroanalytical Chemistry*.
55. J. Jung, L. J. Y. Kim, X. Wang, Q. Wu, T. Sanvoranart, C. G. Hubert, B. C. Prager, L. C. Wallace, X. Jin, S. C. Mack, J. N. Rich, Nicotinamide metabolism regulates glioblastoma stem cell maintenance. *JCI Insight* **2**, e90019 (2017).
56. J.-B. Hövener, A. N. Pravdivtsev, B. Kidd, C. R. Bowers, S. Glöggler, K. V. Kovtunov, M. Plaumann, R. Katz-Brull, K. Buckenmaier, A. Jerschow, F. Reineri, T. Theis, R. V. Shchepin, S. Wagner, P. Bhattacharya, N. M. Zacharias, E. Y. Chekmenev, Parahydrogen-based hyperpolarization for biomedicine. *Angew. Chem. Int. Ed.* **57**, 11140–11162 (2018).
57. J. Milani, B. Vuichoud, A. Bornet, R. Melzi, S. Jannin, G. Bodenhausen, Hyperpolarization of nitrogen-15 nuclei by cross polarization and dissolution dynamic nuclear polarization. *Rev. Sci. Inst.* **88**, 015109 (2017).
58. S. Jannin, A. Bornet, S. Colombo, G. Bodenhausen, Low-temperature cross polarization in view of enhancing dissolution dynamic nuclear polarization in NMR. *Chem. Phys. Lett.* **517**, 234–236 (2011).
59. A. Ferrari, J. Peters, M. Anikeeva, A. Pravdivtsev, F. Ellermann, K. Them, O. Will, E. Peschke, H. Yoshihara, O. Jansen, J.-B. Hövener, Performance and reproducibility of ^{13}C and ^{15}N hyperpolarization using a cryogen-free DNP polarizer. *Sci. Rep.* **12**, 11694 (2022).
60. L. Lumata, M. E. Merritt, C. R. Malloy, A. D. Sherry, Z. Kovacs, Impact of Gd^{3+} on DNP of [1-^{13}C]pyruvate doped with trityl OX063, BDPA, or 4-oxo-TEMPO. *J. Phys. Chem. A* **116**, 5129–5138 (2012).
61. C. Griesinger, M. Bennati, H. M. Vieth, C. Luchinat, G. Parigi, P. Höfer, F. Engelke, S. J. Glaser, V. Denysenkov, T. F. Prisner, Dynamic nuclear polarization at high magnetic fields in liquids. *Prog. Nucl. Magn. Reson. Spectrosc.* **64**, 4–28 (2012).
62. J. R. Birchall, M. S. H. Kabir, O. G. Salnikov, N. V. Chukanov, A. Svyatova, K. V. Kovtunov, I. V. Koptyug, J. G. Gelovani, B. M. Goodson, W. Pham, E. Y. Chekmenev, Quantifying the effects of quadrupolar sinks via ^{15}N relaxation dynamics in metronidazoles hyperpolarized via SABRE-SHEATH. *Chem. Commun.* **56**, 9098–9101 (2020).
63. T. R. Eykyn, P. W. Kuchel, Scalar couplings as pH probes in compartmentalized biological systems: ^{31}P NMR of phosphite. *Magn. Reson. Med.* **50**, 693–696 (2003).
64. A. N. Pravdivtsev, F. D. Sönnichsen, J.-B. Hövener, In vitro singlet state and zero-quantum encoded magnetic resonance spectroscopy: Illustration with *N*-acetyl-aspartate. *PLOS ONE* **15**, e0239982 (2020).
65. A. S. Kiryutin, A. N. Pravdivtsev, K. L. Ivanov, Y. A. Grishin, H.-M. Vieth, A. V. Yurkovskaya, A fast field-cycling device for high-resolution NMR: Design and application to spin relaxation and hyperpolarization experiments. *J. Magn. Reson.* **263**, 79–91 (2016).
66. D. F. Hansen, Measurement of ^{15}N longitudinal relaxation rates in $^{15}NH_4^+$ spin systems to characterise rotational correlation times and chemical exchange. *J. Magn. Reson.* **279**, 91–98 (2017).
67. P. J. Rayner, M. J. Burns, A. M. Olaru, P. Norcott, M. Fekete, G. G. R. Green, L. A. R. Highton, R. E. Mewis, S. B. Duckett, Delivering strong ^{1}H nuclear hyperpolarization levels and long magnetic lifetimes through signal amplification by reversible exchange. *Proc. Natl. Acad. Sci. U.S.A.* **114**, E3188–E3194 (2017).
68. N. V. Chukanov, B. E. Kidd, L. M. Kovtunova, V. I. Bukhtiyarov, R. V. Shchepin, E. Y. Chekmenev, B. M. Goodson, K. V. Kovtunov, I. V. Koptyug, A versatile synthetic route to the preparation of ^{15}N heterocycles. *J. Label. Compd. Radiopharm.* **62**, 892–902 (2019).
69. T. Siber, V. Bušić, D. Zobundžija, S. Roca, D. Vikić-Topić, K. Vranděić, D. Gašo-Sokǎ, An improved method for the quaternization of nicotinamide and antifungal activities of its derivatives. *Molecules* **24**, 1001 (2019).
70. E. I. Slyusarenko, N. P. Gorodetskova, G. V. Pesotskaya, E. S. Levchenko, S. E. Mogilevich, V. D. Luk'yanchuk, Pyridine derivatives possessing hypoglycemic and analgesic activity. *Pharm. Chem. J.* **23**, 739–743 (1989).
71. M. J. van Haren, J. Sastre Toraño, D. Sartini, M. Emanuelli, R. B. Parsons, N. I. Martin, A rapid and efficient assay for the characterization of substrates and inhibitors of nicotinamide *N*-methyltransferase. *Biochemistry* **55**, 5307–5315 (2016).
72. K. Shabalin, K. Nerinovski, A. Yakimov, V. Kulikova, M. Svetlova, L. Solovjeva, M. Khodorkovskiy, S. Gambaryan, R. Cunningham, M. E. Migaud, M. Ziegler, A. Nikiforov, NAD metabolome analysis in human cells using ^{1}H NMR spectroscopy. *Int. J. Mol. Sci.* **19**, 3906 (2018).
73. L. S. Dietrich, L. Fuller, I. L. Yero, L. Martinez, Nicotinamide mononucleotide pyrophosphorylase activity in animal tissues. *J. Biol. Chem.* **241**, 188–191 (1966).
74. S.-I. Imai, The NAD World 2.0: The importance of the inter-tissue communication mediated by NAMPT/NAD^+/SIRT1 in mammalian aging and longevity control. *Npj Syst. Biol. Appl.* **2**, 16018 (2016).
75. J. R. Revollo, A. Körner, K. F. Mills, A. Satoh, T. Wang, A. Garten, B. Dasgupta, Y. Sasaki, C. Wolberger, R. R. Townsend, J. Milbrandt, W. Kiess, S.-I. Imai, Nampt/PBEF/Visfatin regulates insulin secretion in β cells as a systemic NAD biosynthetic enzyme. *Cell Metab.* **6**, 363–375 (2007).
76. A. Grozio, K. F. Mills, J. Yoshino, S. Bruzzone, G. Sociali, K. Tokizane, H. C. Lei, R. Cunningham, Y. Sasaki, M. E. Migaud, S.-I. Imai, Slc12a8 is a nicotinamide mononucleotide transporter. *Nat. Metab.* **1**, 47–57 (2019).
77. Ł. Mateuszuk, R. Campagna, B. Kutryb-Zając, K. Kuś, E. M. Słominska, R. T. Smolenski, S. Chlopicki, Reversal of endothelial dysfunction by nicotinamide mononucleotide via extracellular conversion to nicotinamide riboside. *Biochem. Pharmacol.* **178**, 114019 (2020).
78. C. Zhou, J. Feng, J. Wang, N. Hao, X. Wang, K. Chen, Design of an in vitro multienzyme cascade system for the biosynthesis of nicotinamide mononucleotide. *Cat. Sci. Technol.* **12**, 1080–1091 (2022).
79. J. Hoshino, U. Kühne, H. Kröger, Enhancement of DNA synthesis and cell proliferation by 1-methylnicotinamide in rat liver cells in culture: Implication for its in vivo role. *Biochem. Biophys. Res. Commun.* **105**, 1446–1452 (1982).

80. M. K. Kilgour, S. MacPherson, L. G. Zacharias, A. E. Ellis, R. D. Sheldon, E. Y. Liu, S. Keyes, B. Pauly, G. Carleton, B. Allard, J. Smazynski, K. S. Williams, P. H. Watson, J. Stagg, B. H. Nelson, R. J. DeBerardinis, R. G. Jones, P. T. Hamilton, J. J. Lum, 1-Methylnicotinamide is an immune regulatory metabolite in human ovarian cancer. *Sci. Adv.* **7**, eabe1174 (2021).
81. C. Watała, P. Kaźmierczak, M. Dobaczewski, T. Przygodzki, M. Bartuś, M. Łomnicka, E. M. Słomińska, Z. Durăkova, S. Chłopicki, Anti-diabetic effects of 1-methylnicotinamide (MNA) in streptozocin-induced diabetes in rats. *Pharmacol. Rep.* **61**, 86–98 (2009).
82. N. Singh, A. Miner, L. Hennis, S. Mittal, Mechanisms of temozolomide resistance in glioblastoma—A comprehensive review. *Cancer Drug Resist.* **4**, 17–43 (2021).
83. O. Warburg, On the origin of cancer cells. *Science* **123**, 309–314 (1956).
84. R. E. Hurd, Y.-F. Yen, A. Chen, J. H. Ardenkjaer-Larsen, Hyperpolarized ^{13}C metabolic imaging using dissolution dynamic nuclear polarization. *J. Magn. Reson. Imaging* **36**, 1314–1328 (2012).
85. C. A. Müller, C. Hundshammer, M. Braeuer, J. G. Skinner, S. Berner, J. Leupold, S. Düwel, S. G. Nekolla, S. Månsson, A. E. Hansen, D. von Elverfeldt, J. H. Ardenkjaer-Larsen, F. Schilling, M. Schwaiger, J. Hennig, J.-B. Hövener, Dynamic 2D and 3D mapping of hyperpolarized pyruvate to lactate conversion in vivo with efficient multi-echo balanced steady-state free precession at 3 T. *NMR Biomed.* **33**, e4291 (2020).
86. J. Singh, E. H. Suh, G. Sharma, C. Khemtong, A. D. Sherry, Z. Kovacs, Probing carbohydrate metabolism using hyperpolarized ^{13}C-labeled molecules. *NMR Biomed.* **32**, e4018 (2018).
87. A. Eldirdiri, A. Clemmensen, S. Bowen, A. Kjær, J. H. Ardenkjær-Larsen, Simultaneous imaging of hyperpolarized [1,4-$^{13}C_2$]fumarate, [1-^{13}C]pyruvate and ^{18}F-FDG in a rat model of necrosis in a clinical PET/MR scanner. *NMR Biomed.* **30**, e3803 (2017).
88. H. A. I. Yoshihara, J. A. M. Bastiaansen, M. Karlsson, M. H. Lerche, A. Comment, J. Schwitter, Detection of myocardial medium-chain fatty acid oxidation and tricarboxylic acid cycle activity with hyperpolarized [1–^{13}C]octanoate. *NMR Biomed.* **33**, e4243 (2020).
89. X. Ji, A. Bornet, B. Vuichoud, J. Milani, D. Gajan, A. J. Rossini, L. Emsley, G. Bodenhausen, S. Jannin, Transportable hyperpolarized metabolites. *Nat. Commun.* **8**, 13975 (2017).
90. A. N. Pravdivtsev, G. Buntkowsky, S. B. Duckett, I. V. Koptyug, J.-B. Hövener, Parahydrogen-induced polarization of amino acids. *Angew. Chem. Int. Ed.* **60**, 23496–23507 (2021).
91. D. O. Guarin, S. M. Joshi, A. Samoilenko, M. S. H. Kabir, E. E. Hardy, A. M. Takahashi, J. H. Ardenkjaer-Larsen, E. Y. Chekmenev, Y.-F. Yen, Development of dissolution dynamic nuclear polarization of [$^{15}N_3$]metronidazole: A clinically approved antibiotic. *Angew. Chem. Int. Ed.* **62**, e202219181 (2023).
92. J. H. Ardenkjær-Larsen, S. Bowen, J. R. Petersen, O. Rybalko, M. S. Vinding, M. Ullisch, N. C. Nielsen, Cryogen-free dissolution dynamic nuclear polarization polarizer operating at 3.35 T, 6.70 T, and 10.1 T. *Magn. Reson. Med.* **81**, 2184–2194 (2019).
93. A. Capozzi, J. Kilund, M. Karlsson, S. Patel, A. C. Pinon, F. Vibert, O. Ouari, M. H. Lerche, J. H. Ardenkjær-Larsen, Metabolic contrast agents produced from transported solid ^{13}C-glucose hyperpolarized via dynamic nuclear polarization. *Commun. Chem.* **4**, 95 (2021).
94. K. Hattermann, J. Held-Feindt, R. Lucius, S. S. Müerköster, M. E. T. Penfold, T. J. Schall, R. Mentlein, The chemokine receptor CXCR7 is highly expressed in human glioma cells and mediates antiapoptotic effects. *Cancer Res.* **70**, 3299–3308 (2010).
95. D. Caylioglu, R. J. Meyer, D. Hellmold, C. Kubelt, M. Synowitz, J. Held-Feindt, Effects of the anti-tumorigenic agent AT101 on human glioblastoma cells in the microenvironmental glioma stem cell niche. *Int. J. Mol. Sci.* **22**, 3606 (2021).
96. J. Held-Feindt, S. Schmelz, K. Hattermann, R. Mentlein, H. M. Mehdorn, S. Sebens, The neural adhesion molecule L1CAM confers chemoresistance in human glioblastomas. *Neurochem. Int.* **61**, 1183–1191 (2012).
97. J. Ratajczak, M. Joffraud, S. A. J. Trammell, R. Ras, N. Canela, M. Boutant, S. S. Kulkarni, M. Rodrigues, P. Redpath, M. E. Migaud, J. Auwerx, O. Yanes, C. Brenner, C. Cantó, NRK1 controls nicotinamide mononucleotide and nicotinamide riboside metabolism in mammalian cells. *Nat. Commun.* **7**, 13103 (2016).
98. H. Okamoto, A. Ishikawa, Y. Yoshitake, N. Kodama, M. Nishimuta, T. Fukuwatari, K. Shibata, Diurnal variations in human urinary excretion of nicotinamide catabolites: Effects of stress on the metabolism of nicotinamide. *Am. J. Clin. Nutr.* **77**, 406–410 (2003).
99. Z. Országhová, O. Ulĭná, A. Liptáková, I. Žitňanová, J. Muchová, C. Watala, Z. Ďuráková, Effects of N^1-methylnicotinamide on oxidative and glycooxidative stress markers in rats with streptozotocin-induced diabetes mellitus. *Redox Rep.* **17**, 1–7 (2012).
100. K. L. Bogan, C. Brenner, Nicotinic acid, nicotinamide, and nicotinamide riboside: A molecular evaluation of NAD^+ precursor vitamins in human nutrition. *Annu. Rev. Nutr.* **28**, 115–130 (2008).

Acknowledgments

Funding: We acknowledge funding from German Federal Ministry of Education and Research (BMBF) within the framework of the e:Med research and funding concept (01ZX1915C), DFG (PR 1868/3-1, HO-4602/2-2, HO-4602/3, GRK2154-2019, EXC2167, FOR5042, and TRR287) and financial support by Land Schleswig-Holstein within the funding programme Open Access Publikationsfonds. MOIN CC was funded by a grant from the European Regional Development Fund (ERDF) and the Zukunftsprogramm Wirtschaft of Schleswig-Holstein (project no. 122-09-053). A.N.P. acknowledges Kiel University PMI cluster of excellence for intramural supporting enzymatic LDH activity studies. J.M. received funding from FNR-INTER (NTER/BMBF/18/13399110). We are also thankful to N. Kiweler and L. Neises from LIH for providing us with K-562 cells. **Author contributions:** A.N.P. and J.-B.H.: conceptualization. A.N.P. and J.P.P.: investigation, analysis, and writing—original draft. A.B. and R.H.: synthesis of 1-^{15}N-NAM. V.J., E.P., F.E., and M.A.: design and construction of ^{15}N-probe. J.P.P., A.F., and A.N.P.: DNP studies. A.N.P., J.P.P., and E.P.: ^{15}N MRI. J.H.-F., D.H., N.-m.K., K.A., and J.M.: cell cultivation and metabolic-model study. A.N.P. and J.-B.H.: supervision and funding acquisition. All authors contributed to discussions and interpretation of the results and have approved the final version of the manuscript. **Competing interests:** The authors declare that they have no competing interests. **Data and materials availability:** All data needed to evaluate the conclusions in the paper are present in the paper and/or the Supplementary Materials. The corresponding raw data can be accessed via Zenodo DOI: 10.5281/zenodo.8171191.

Submitted 17 May 2023
Accepted 21 July 2023
Published 23 August 2023
10.1126/sciadv.add3643
