# Supplementary material for: Nitrogen-15 dynamic nuclear polarization of nicotinamide derivatives in biocompatible solutions
Source: Sci Adv. 2023 Aug 23;9(34):eadd3643. doi: 10.1126/sciadv.add3643 (PMC10446501; doi:10.1126/sciadv.add3643)
Supplement: Supplementary file 2 — Sections S1 to S18 Figs. S1 to S15 Tables S1 and S2 [file sciadv.add3643_sm.pdf]

Science Advances
AAAS

Supplementary Materials for

Nitrogen-15 dynamic nuclear polarization of nicotinamide derivatives in biocompatible solutions

Josh P. Peters *et al.*

Corresponding author: Jan-Bernd Hövener, jan.hoevener@rad.uni-kiel.de;
Andrey N. Pravdivtsev, andrey.pravdivtsev@rad.uni-kiel.de

Sci. Adv. **9**, eadd3643 (2023)
DOI: 10.1126/sciadv.add3643

This PDF file includes:

Sections S1 to S18
Figs. S1 to S15
Tables S1 and S2

1. Sample preparation and composition

The dDNP sample preparation and dissolution media are described in the main text. If the sample's composition is different, then it is detailed together with the experimental data. The complete list of samples is given in **Table S1**.

Sample polarization and solid-state build-up time, $T_{\text{b-up}}$, as a function of radical concentration for two glassing agents, is given in **Figure S1**.

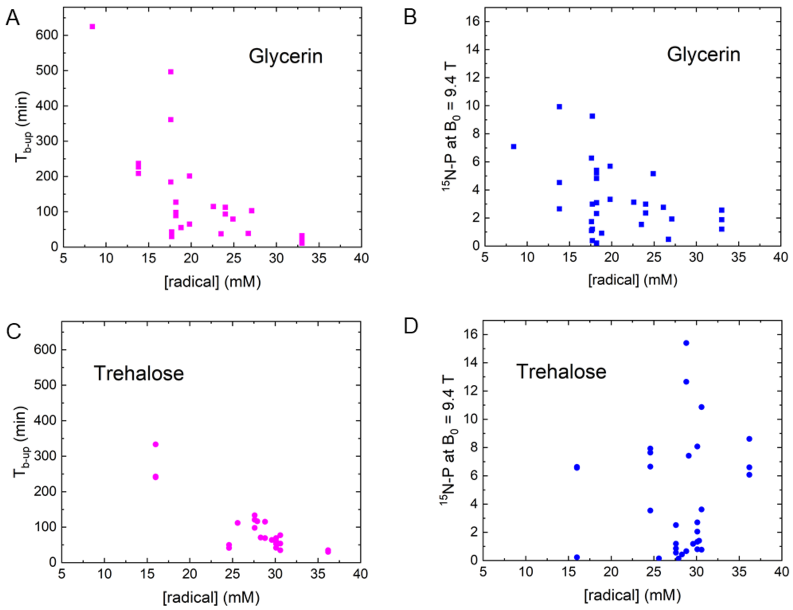

Figure S1. Solid state polarization build-up time and liquid state ^{15}N polarization (measured at 9.4 T and basic pH. *Build-up time, $T_{b\text{-}up}$, (A,C) and liquid state ^{15}N polarization (B,D) were measured as a function of trityl radical concentration when glycerol (A,B) or trehalose (C,D) were used as a glassing agent. Using trehalose, radical concentrations of around 30 mM seem to be best to achieve high ^{15}N polarizations of >10% within a couple of hours. Unfortunately, the experiments lasted over 12 months, resulting in lower reproducibility. But general conclusions can still be drawn.*

Table S1. Complete list of the dDNP samples used in this study. *The amount of water, ^{15}N-NAM, amount of radical and its type, amount and type of glassing agent, and the concentration of gadolinium contrast agent are given.*

Mixture ID	H_2O [mg]	NAM mg	NAM [mol/L]	Chemical	NAM chemical ID	Radical [mg]	Radical [mmol/L]	Radical type	Glassing agent (GA)	GA [mg]	GA [mol/L]	Gadolinium [mmol/L]	Sample volume [µL]
**5**	201.7	203.7	3.65	1-15N-nicotinamide	A - Batch 1	13.3	18.2	AH111501	Glycerin	73.4	1.74	0.0	457
**6**	180.2	200.4	3.70	1-15N-nicotinamide	A - Batch 1	14.0	19.8	AH111501	Glycerin	95.1	2.33	0.0	443
**7**	200.1	195.6	3.46	1-15N-nicotinamide	A - Batch 1	13.0	17.6	AH111501	Glycerin	94.9	2.23	0.0	463
**8**	119.6	103.3	3.38	1-15N-nicotinamide	A - Batch 1	8.9	22.3	AH111501	Glycerin	48.4	2.10	0.0	250
**9**	119.5	102.1	3.34	1-15N-nicotinamide	B - Batch 1	7.9	19.8	AH111501	Glycerin	53.0	2.30	0.0	250
**10**	122.0	102.2	3.35	1-15N-nicotinamide	B - Batch 1	9.8	24.6	AH111501	Glycerin	52.6	2.28	0.0	250
**11**	31.6	101.7	2.97	1-15N-nicotinamide	B - Batch 1	11.1	24.9	AH111501	Glycerin	51.1	1.98	0.0	280
**12**	149.8	116.4	3.53	1-15N-nicotinamide	B - Batch 1	11.5	26.7	AH111501	Glycerin	35.0	1.41	0.0	270
**13**	121.4	99.5	3.26	1-15N-nicotinamide	C - Batch 1	9.0	22.6	AH111501	Glycerin	49.7	2.16	0.0	250
**14**	137.0	117.4	3.43	1-15N-nicotinamide	C - Batch 1	10.7	24.0	AH111501	Glycerin	58.8	2.28	1.0	280
**15**	64.0	53.4	3.50	1-15N-nicotinamide	A - Batch 1	5.4	27.1	AH111501	Glycerin	28.6	2.48	0.0	125
**16**	120.0	101.0	3.68	1-15N-nicotinamide	TS202B 18.05.22	9.9	27.6	AH111501	Trehalose	71.1	0.92	0.0	225
**17**	99.4	100.4	3.82	1-15N-nicotinamide	TS202B 18.05.22	10.0	29.2	AH111501	Trehalose	62.0	0.84	0.5	215
**18**	100.1	100.7	3.84	1-15N-nicotinamide	TS202B 18.05.22	10.3	30.0	AH111501	Trehalose	55.5	0.75	0.5	215
**19**	98.3	101.1	3.85	1-15N-nicotinamide	TS202B 18.05.22	9.4	27.4	AH111501	Trehalose	62.1	0.84	0.5	215
**20**	101.0	99.5	3.33	1-15N-nicotinamide	TS202B 18.05.22	10.6	27.1	AH111501	Trehalose	60.5	0.72	0.5	245
**21**	110.0	100.5	3.36	1-15N-nicotinamide	TS202B 18.05.22	10.9	27.9	AH111501	Trehalose	69.1	0.82	0.5	245
**22**	111.8	101.1	3.37	1-15N-nicotinamide	TS202B 18.05.22	11.3	28.8	AH111501	Trehalose	70.6	0.84	0.5	246
**23**	109.4	99.9	3.33	1-15N-nicotinamide	TS202B 18.05.22	12.0	30.6	AH111501	Trehalose	70.0	0.83	0.5	246
**24**	221.8	199.5	3.32	1-15N-nicotinamide	TS202B (90.6), TS202A (109.5)	23.8	30.3	AH111501	Trehalose	142.3	0.84	0.5	492
**25**	110.7	100.6	3.35	1-15N-nicotinamide	TS202A	11.4	32.5	OX063	Trehalose	73.4	0.87	0.0	246
**26**	110.1	103.0	3.43	1-15N-nicotinamide	TS202A	14.2	40.5	OX063	Trehalose	69.2	0.82	0.0	246
**27**	129.6	100.1	3.04	1-15N-nicotinamide	19/12/2022	10.6	24.6	AH111501	Trehalose	68.0	0.74	0.0	270
**28**	152.0	110.9	3.36	1-15N-nicotinamide	19/12/2022	12.2	28.2	AH111501	Trehalose	68.0	0.74	0.5	270
**29**	129.8	102.8	3.58	14N-nicotinamide	Sigma 14N CAS: 98-92-0	5.9	15.7	AH111501	Trehalose	52.3	0.65	0.0	235
**30**	140.4	111.4	3.88	1-15N-nicotinamide	19/12/2022	6.0	16.0	AH111501	Trehalose	63.6	0.79	6.2	235
**31**	63.1	53.7	3.74	1-15N-nicotinamide	19/12/2022	1.2	62.6	TEMPO	Trehalose	37.5	0.93	0.0	117.5
**32**	127.8	109.5	3.82	1-15N-nicotinamide	19/12/2022	11.1	29.7	AH111501	Trehalose	78.2	0.97	0.7	235
**33**	129.5	101.8	3.55	1-15N-nicotinamide	19/12/2022	6.0	16.0	AH111501	Trehalose	79.5	0.99	0.7	235
**34**	133.8	112.5	3.84	1-15N-nicotinamide	19/12/2022	11.3	29.5	AH111501	Trehalose	78.4	0.95	0.6	240
**35**	129.6	108.4	3.62	1-15N-nicotinamide	19.12.2022 & 02.03.2023	10.5	26.9	AH111501	Trehalose	92.9	1.11	0.0	245
**36**	119.4	97.5	3.26	1-15N-nicotinamide	02/03/2023	10.6	27.1	AH111501	Trehalose	104.6	1.25	0.0	245
**37**	130.1	110.2	3.84	1-15N-nicotinamide	14/03/2023	10.6	28.3	AH111501	Trehalose	50.5	0.63	0.0	235
**38**	120.3	97.6	3.63	1-15N-nicotinamide	14/03/2023	10.9	31.1	AH111501	Trehalose	35.4	0.47	0.0	220
**39**	130.5	99.6	3.33	1-15N-nicotinamide	14/03/2023	10.9	27.8	AH111501	Trehalose	63.8	0.76	0.0	245
**40**	119.5	100.2	3.73	1-15N-nicotinamide	14/03/2023	11.0	31.3	AH111501	Trehalose	58.4	0.78	0.0	220
**41**	129.0	100.2	3.65	1-15N-nicotinamide	14/03/2023	10.5	29.3	AH111501	Trehalose	53.4	0.69	0.0	225
**42**	129.0	101.9	3.71	1-15N-nicotinamide	14/03/2023	10.1	28.0	AH111501	Trehalose	61.3	0.80	0.0	225
**43**	129.4	101.1	3.68	1-15N-nicotinamide	14/03/2023	10.2	28.4	AH111501	Trehalose	62.3	0.81	0.0	225

2. Synthesis of 1-^{15}N-NAM

The synthesis of 1-^{15}N-NAM repeats the methods proposed before(Ref 40, Shchepin et al) with deviation in purification. 1-^{15}N-NAM was synthesized in a two-step reaction from nicotinamide (NAM, 72340, CAS: 98-92-0, Sigma-Aldrich) via the Zincke salt followed by nitrogen exchange with $^{15}NH_4Cl$ (299251, CAS: 39466-62-1, Sigma-Aldrich). In the first step, the Zincke salt of NAM is formed with 1-chloro-2,4-dinitrobenzene (237329, CAS: 97-00-7, Sigma-Aldrich) in DMSO. The resulting compound was a slightly yellow powder, which was subsequently reacted with $^{15}NH_4Cl$ to obtain 1-^{15}N-NAM as a white powder. As a result, we achieved a 40% yield of ^{15}N-NAM with 91 ± 2 % ^{15}N enrichment and 99% purity according to ^{1}H NMR. Below we will detail essential steps and pitfalls in the synthesis.

Step 1. Synthesis of the Zincke salt

5.25 g (42.9 mmol) NAM was dissolved together with 20.00 g (98.74 mmol) 1-chloro-2,4-dinitrobenzene in 10 ml of dry DMSO. To dissolve all educts faster, the suspension was carefully warmed up with a heat gun to approximately 40°C until all solid components were wholly dissolved. The resulting solution was stirred for five days at room temperature. Then it was slowly poured into 90 mL of dry acetone under vigorous stirring. We recommend using a funnel with a small outlet to pour the highly viscous DMSO solution into the acetone under vigorous stirring. Only then a finely powdered precipitate can be obtained and can be conveniently used further. The acetone was decanted carefully, and another portion of 70 mL of dry acetone was added to wash the precipitate. The acetone washing was repeated three times more. After the last washing, the remaining acetone was removed i. vac. The slightly yellow residue was dissolved in 100 mL of dry methanol and stored in a sealed flask under a nitrogen atmosphere.

Step 2. Exchange with $^{15}NH_4Cl$

A 2 L three-neck flask flushed with nitrogen was charged 6.75 g (124 mmol) ^{15}N-ammonium chloride and 1.5 mL freshly dried methanol. The solution was cooled to 0°C, and 6.20 g (115 mmol) of sodium methanolate solved in 25 mL methanol was added dropwise. After stirring for 15 min at 0°C, the previously prepared methanolic solution of Zincke salt was added dropwise over a dropping funnel. The reaction was allowed to warm to room temperature and was stirred for another two days.

Step 3. Purification of the product

Activated carbon was added and filtered shortly after. The solvent was then removed, i. vac., and the residue was resolved in HPLC-grade water. Another portion of activated carbon was added and filtrated shortly after, and the solvent was removed, i. vac. This step was repeated at pH 12 and then at pH 6. In the end, the residue was purified via flash-chromatography using 10:1 ethyl acetate:methanol solution to obtain 2.08 g (17.0 mmol) of 1-^{15}N-NAM as a white powder. We note that the addition of activated charcoal is essential for purification. Otherwise, NAM could not be chromatographically separated from the intensely colored, yellow side product 2,4-dinitroaniline. As a result, the product sometimes had a yellow tint after the chromatographic workup. In these cases, the raw product was recrystallized from ethyl acetate. We found that the recrystallization out of 1-pentanone(Ref 40, Shchepin et al) is unsuitable for 1-^{15}N-NAM.

3. Spontaneous conversion of NAM to NA (thermal polarization)

To study the conversion rate of NAM to NA, we prepared two samples: The initial composition of the first sample was an aqueous solution (with 10% D_2O for lock) of 0.25 M NAM at a pH of 13.66. A conversion of around 80% (NAM to NA) at 335 K of the initially present NAM was observed within one hour **(Figure S2**). However, the conversion was limited by the available ^-OH. So, for the second sample with the initial concentration of NAM of 1 M, only a quarter of the available NAM was converted (not shown here). Note that the solubility of NA is much lower than that of NAM, which should also be considered.

In the main text, we report NAM to NA conversion before dDNP (**Figure 5**). This was achieved with the following sample composition: 32 mg water, 51 mg glycerol, 102 mg of 1-^{15}N-NAM, 11.1 mg trityl radical, and 152 mg of 1 M NaOH with the final pH of 13.33.

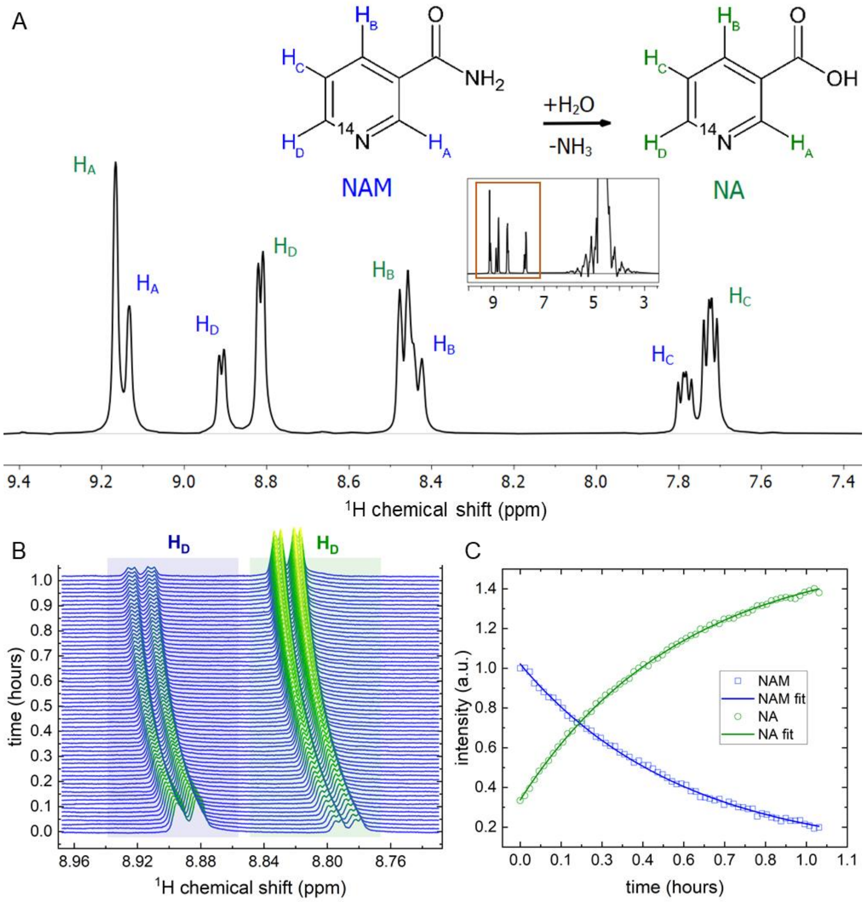

Figure S2. Conversion of NAM to NA at a basic aqueous solution. *(A) Scheme of the chemical reaction and ^{1}H NMR spectrum of NAM and NA in water at a pH of 13.7. The red square shows the enlarged area of the spectrum. One can see the H_A, H_D, H_B, and H_C spectral lines of NAM and NA from left to right on the spectrum. The H_D lines of NAM and NA do not overlap; therefore, they were chosen for the kinetic analysis. (B) H_D signals of NAM and NA as a function of experiment number (time). The chemical shift was changing due to a decrease in pH throughout the reaction. Each spectrum was measured with NS = 1 and TR = 58 s. (C) NAM to NA conversion is demonstrated by integral values of H_D signals of NAM (blue) and NA (green) as a function of time. The two kinetics were fitted with the exponential decay functions in the form of $A + Be^{-t/T}$ with the shared time-constant T of (33.0±0.4) min and conversion rate w of (1.82±0.02)·h^{-1}.*

4. Hyperpolarization of the amide (NH_2) of NAM

In some experiments, natural abundance ^{15}N-amide was polarized (**Figure S3, and Figure 3A spectrum 4**). We used the same Eq. 1 from the main script with a slight modification to estimate achieved polarization. Because we do not see ^{15}N-amide in the spectrum at thermal equilibrium, we estimated it via a thermal signal of 1-^{15}N. The natural abundance of ^{15}N is 0.3%, while the ^{15}N enrichment of 1-^{15}N was over 91%. Then the hyperpolarized signal of ^{15}N-amide should be compared with the 1-^{15}N signal at thermal equilibrium using Eq. 1, and the result for polarization should be multiplied by 91/0.3 = 303. This way, we estimated the polarization of ^{15}N-amide to be 13.6% at the time of measurement. This approximation was supported by the DNP of a natural abundance NAM sample, which led to a similar ^{15}N-amide signal and a similar polarization approximation for 1-^{15}N-NAM.

T_1 for ^{15}N-amide was found to be much longer in D_2O and even at neutral pH, with T_1 of 51.8 s and higher at pH 7.5 and 9.4 T. This is 3-4 times longer compared to 1-^{15}N-NAM under similar conditions.

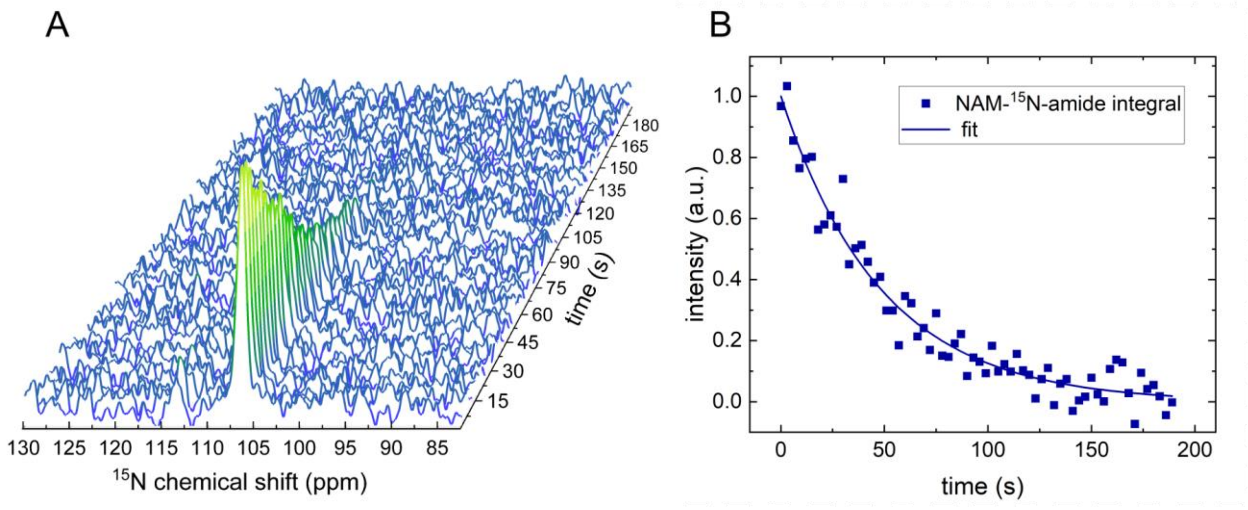

Figure S3. ^{15}N Hyperpolarized spectra and signal decay of the ^{15}N-amide of 1-^{15}N-NAM-^{15}N-amide. *Signal decay (A) and corresponding integrals (B) were observed using TR = 3 s. Integrals as a function of excitation time (squares) were fitted using a mono-exponential decay function, giving a corrected signal decay (Eq. 3, main text) of 51.8 s. Using the ^{15}N spectrum of the amide side of NAM, we estimated polarization to be 13.6% at the time of measurement (t = 28 s after dissolution) with an SNR of 8.9. No hyperpolarized 1-^{15}N-NAM signal and no ^{15}N-amide signal at thermal equilibrium was observed. The dDNP sample composition was: 100 mg water, 56 mg trehalose, 101 mg of 1-^{15}N-NAM, and 10.3 mg trityl radical. The pH value of the dissolved sample with deuterated neutral-DM was 7.45.*

5. Effect of amount of dissolution media on liquid state T_1

We found that varying the amount of DM used for dissolution led to a significant change in liquid state T_1 when measured at 1 T (**Table S2, Figure S4**). This may be due to different concentrations of dissolved ions or oxygen in the hyperpolarized solution. Ions can be introduced by corrosion in the dissolution chamber or the dissolution pathway. It was found that this T_1 variation is not dependent on NAM or radical concentration but on the amount of dissolution media only.

Table S2: Effect of DM amount on sample T_1. *A strong dependency of DM amount on sample T_1 can be observed for hyperpolarized NAM. T_1 using 8 mL DM was found to be improved by over 56 % compared to 4 mL. Experiments 120 and 121 suggest, that this is not an effect of [NAM] or [Trityl], since final concentrations are about the same and only thing different is the amount of DM. Note that the sample composition is the same, and the weight of the sample is proportional to the final liquid state concentration for all samples.*

#sample	Sample weight [mg]	DM [mL]	T1 @1T [s]
106	58.0	4	32.2
107	57.4	4	33.3
116	59.5	4	42.1
120	29.2	4	35.4
		**Average:**	**35.8 ± 3.8**
117	55.7	8	59.9
118	58.4	8	54.7
119	58.5	8	53.3
121	57.9	8	55.6
		**Average:**	**55.9 ± 2.5**

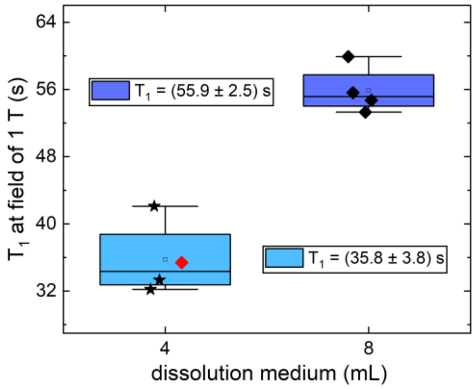

Figure S4. Effect of amount of basic dissolution media on ^{15}N polarization lifetime of 1-^{15}N-NAM. Two sets of four experiments were carried out using 4 and 8 mL of basic-DM, respectively. A statistically significant difference of 20.1 s was found between the two sets, which were not related to NAM or radical concentrations (one-sided t-test, p = 0.48%). Note that we compared two sets of concentrations after dissolution with 4 or 8 mL: [1-^{15}N-NAM] ≈ 20 mM, and [trytil radical] ≈ 0.15 mM (diamonds) and double concentration (stars). The concentration of NAM and trityl did not change the observed T_1. This suggests that the effect seen is independent of [1-^{15}N-NAM] and [trityl radical]. The small rectangles indicate the mean T_1 (4 observables for each set of 4 mL and 8 mL), the blue boxes indicate the 25 and 75 percentiles, the horizontal line indicates the 50 percentile, and the whiskers indicate the upper and lower values.

6. Neutralization of ^{15}N-NAM

After dissolution (and pH neutralization if applied), we measured the lifetime of ^{15}N polarization at 7 T and 9.4 T as a function of pH (**Figure S5**). The longest lifetime of 1-^{15}N-NAM was measured at basic pH. However, a pH of 8 to 8.5 also seems sufficient for long T_1 ~20 to 30 s. Note the phase separation in **Figure S6** due to pH, which can be observed in the hyperpolarized spectrum (**Figure 3A spectrum 2**, main text).

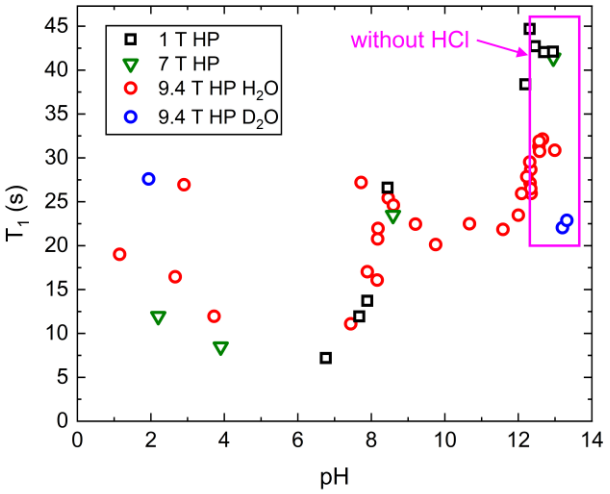

Figure S5. The lifetime of ^{15}N polarization at liquid-state of hyperpolarized 1-^{15}N-NAM. *Reproducible HP T_1 values were obtained after dissolution with basic-DM. However, when HCl was added after the dissolution to neutralize the hyperpolarized sample, high variability in T_1 values, independent of pH value, was observed. Although the values at low pH are not reproducible, there is a tentative trend of higher T_1 values at extreme pH (closer to 0 or 14) and lower T_1 for samples dissolved in D_2O. Note a peak in T_1 at around pH 8.5, which can be exploited for* in vivo *applications.*

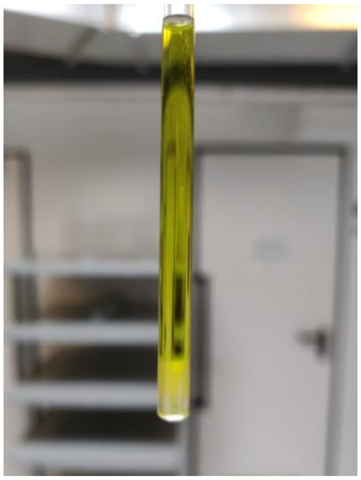

Figure S6. Dissolved 1-^{15}N-NAM sample after dDNP and neutralization attempt. *Insufficient shaking led to inadequate mixing, resulting in the formation of two separate phases: one acidic at pH 0.3 and one basic at pH 12.7.The acidic phase led to (partial) precipitation of the radical and maybe other ingredients. This sample measured at 9.4 T can be seen in* ***Figure 3A spectrum 2****.*

7. Effects of pH on chemical shift of 1-^{15}N-NAM

To investigate the influence of pH on NAM structure we measured ^{1}H and ^{15}N NMR spectra of 800 mM 1-^{15}N-NAM sample in H_2O: D_2O = 9:1 in thermal equilibrium as a function of pH (**Figure S7**). pH was varied by adding 30% HCl and 40% NaOH directly in the NMR tube. A change in the ^{15}N chemical shift in proximity to the NAM pKa of 3.35 (at 20°)(Ref 58, Perrin et al) was observable. The speed of chemical shift change was around 38 ppm/pH unit near the pKa value.

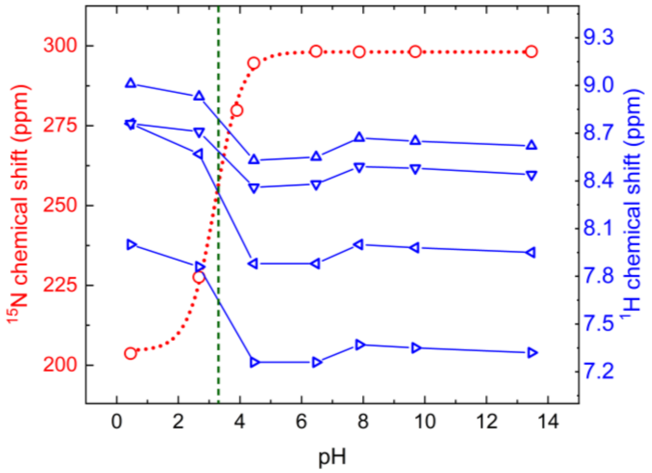

Figure S7. Chemical shift of 1-^{15}N-NAM as a function of pH at T=298 K. *The chemical shift of ^{15}N (red) and ^{1}H (blue) at different pH values of the same consisted of 0.8 M 1-^{15}N-NAM in H_2O:D_2O = 9:1 measured at 9.4 T, 298 K and varying pH. ^{1}H chemical shift was locked at 4.7 ppm for H_2O during the whole duration of the experiment for reference. A strong change in the ^{15}N chemical shift for 1-^{15}N-NAM can be observed between pH 1 and 5, corresponding well with the observed pKa value of 3.2 at room temperature (green dashed line). The Henderson-Hasselbalch equation (Eq. S9, red dotted line on B) was fit to the chemical shifts to obtain the pKa and chemical shift limits under acidic (protonated NAM or HNAM) and basic conditions (NAM):* $pKa = 3.2 \pm 0.05$, $\delta_{HNAM} = 204.5 \pm 1.6\ ppm$, $\delta_{NAM} = 298.1 \pm 0.8\ ppm$.

8. Effects of temperature on the lifetime of 1-^{15}N-NAM

To investigate the effect of temperature on T_1, a 0.1 M 1-^{15}N-NAM sample was measured in 600 µL 17 % D_2O solution. The sample was prepared with pure 1-^{15}N-NAM only, leading to pH 7.37 (**Figure S8).** A strong dependency of the ^{15}N-T_1 on temperature was observable. An apparent increase in T_1 was found from 273 K up to 340 K, after which the ^{15}N T_1 decays slightly. The ^{1}H-^{15}N INEPT enhanced ^{15}N signal decay sequence was used.

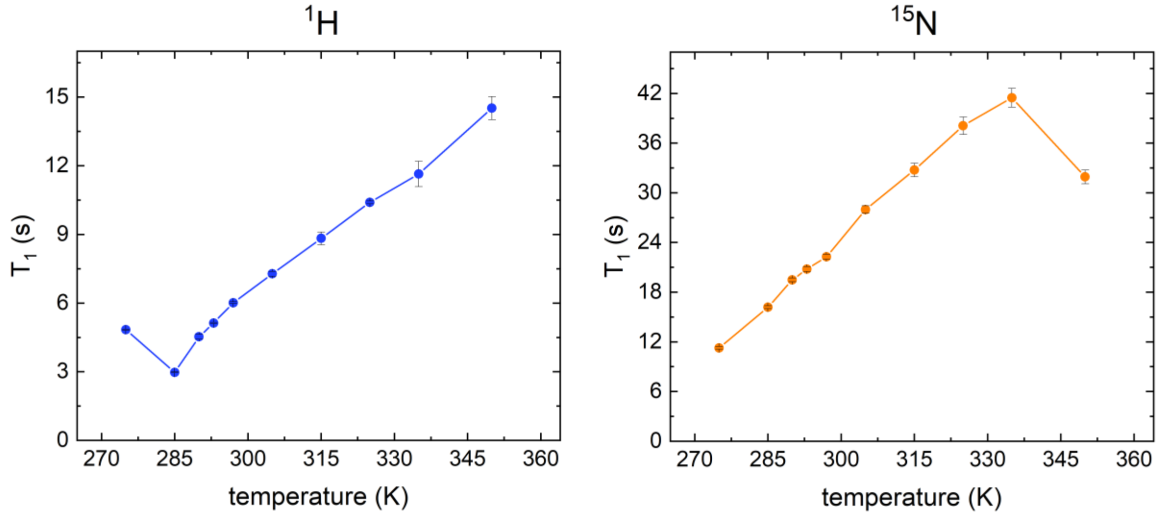

***Figure S8. Relaxation of 1-^{15}N-NAM as a function of temperature at pH 7.37**, revealing a steep increase in T_1 proportional to an increase in temperature. A sudden decrease in T_1 was observed for temperatures above 340 K, which is well above body temperature for* in vivo *studies. Spectra were measured at 9.4 T with a TR = 84 s using the INEPT sequence for increased signal strength. A time of 30 minutes was chosen as a delay between measurements to allow temperature changes to stabilize.*

9. Estimation of chemical exchange rates for the 1-N of NAM

We were unable to observe the protonated NAM species (HNAM) using ^{1}H or ^{15}N NMR spectroscopy. However, the only a single averaged signal between HNAM and NAM was visible instead of two distinct ones: e.g., see chemical shifts of ^{15}N as a function of pH at **Figure S7**. This situation is characteristic for a fast chemical exchange. In this section, we will describe how the lower boundaries for the chemical exchange can be estimated based on this observation.

The following two equations give the chemical exchange of H with NAM:

$$\begin{cases} NAM + H \overset{k_1}{\rightarrow} HNAM \\ HNAM \overset{k_2}{\rightarrow} NAM + H \end{cases} \quad \text{(Eq. S1)}$$

where HNAM is NAM with an additional H on the 1-N site.

Because the concentration of H stays constant, the biomolecular reaction step can be simplified using a pseudo-first-order reaction constant $k_1[H]$ as follows:

$$\begin{cases} NAM \xrightarrow{k_1[H]} HNAM \\ HNAM \overset{k_2}{\rightarrow} NAM + H \end{cases} \quad \text{(Eq. S2)}$$

The evolution of the transversal component of ^{15}N magnetization for NAM and MNAM can be found using a Bloch-McConnell equation:

$$\begin{cases} \frac{d}{dt} M_{tr}^{NAM} \xrightarrow{k_1[H]} (-i\omega_{NAM} - R_2^{NAM}) M_{tr}^{NAM} - k_1[H] M_{tr}^{NAM} + k_2 M_{tr}^{HNAM} \\ \frac{d}{dt} M_{tr}^{HNAM} \overset{k_2}{\rightarrow} (-i\omega_{HNAM} - R_2^{HNAM}) M_{tr}^{HNAM} + k_1[H] M_{tr}^{NAM} - k_2 M_{tr}^{HNAM} \end{cases} \quad \text{(Eq. S3)}$$

Which can also be written in a matrix form as

$$\frac{d}{dt}\begin{pmatrix} M_{tr}^{NAM} \\ M_{tr}^{HNAM} \end{pmatrix} = \begin{pmatrix} -i\omega_{NAM} - R_2^{NAM} - k_1[H] & k_2 \\ k_1[H] & -i\omega_{HNAM} - R_2^{HNAM} - k_2 \end{pmatrix} \begin{pmatrix} M_{tr}^{NAM} \\ M_{tr}^{HNAM} \end{pmatrix} = \hat{A} \begin{pmatrix} M_{tr}^{NAM} \\ M_{tr}^{HNAM} \end{pmatrix} \quad \text{(Eq. S4)}$$

Here ω represents the Larmor precession frequency, R_2 the transversal relaxation rate and $\hat{A}$ the matrix of chemical exchange, relaxation and precession. The initial conditions for magnetization can be found from the chemical equilibrium specific to a chemical reaction (Eq. S1):

$$\frac{[NAM]}{[HNAM]} = \frac{k_2}{k_1[H]} = \frac{M_{tr}^{NAM}(t=0)}{M_{tr}^{HNAM}(t=0)} \quad \text{(Eq. S5)}$$

We will keep the total initial magnetization constant and equal to 1, leading to the initial magnetization amplitudes

$$\begin{cases} M_{tr}^{NAM}(t=0) = \frac{1}{1+\frac{k_2}{k_1[H]}} \\ M_{tr}^{NAM}(t=0) = \frac{\frac{k_2}{k_1[H]}}{1+\frac{k_2}{k_1[H]}} \end{cases} \quad \text{(Eq. S6)}$$

In this case, the solution for the Eq. S4 is given by

$$\begin{pmatrix} M_{tr}^{NAM} \\ M_{tr}^{HNAM} \end{pmatrix}(t) = e^{\hat{A}t} \begin{pmatrix} M_{tr}^{NAM} \\ M_{tr}^{HNAM} \end{pmatrix}(t=0) \quad \text{(Eq. S7)}$$

The concentration [H] in mol/L can be determines from the pH by

$$[\mathrm{H}] = 10^{-\mathrm{pH}} \quad \text{(Eq. S8)}$$

The observed chemical shift, δ_{obs}, as a function of pH can be fitted using the commonly used Henderson-Hasselbalch equation (Ref 67, Eykyn et al; Ref 68, Pravdivtsev et al):

$$\delta_{obs} = \delta_{HNAM} + \frac{\delta_{NAM} - \delta_{HNAM}}{1 + 10^{(\mathrm{pKa} - \mathrm{pH})}} \quad \text{(Eq. S9)}$$

In case of $pH = pKa$, the chemical shift lies exactly in the middle:

$$\delta_{obs}(pH = pKa) = \frac{\delta_{NAM} + \delta_{HNAM}}{2} \quad \text{(Eq. S10)}$$

The position of the resonance frequency under fast chemical exchange is well known and given by the weighted average of the chemical shift of the two corresponding species, NAM and HNAM, as

$$\delta_{obs} = p_{HNAM}\delta_{HNAM} + p_{NAM}\delta_{HNAM} \quad \text{(Eq. S11)}$$

where $p_X = \frac{[X]}{[HNAM]+[NAM]}$ corresponds to the fraction of NAM or HNAM. When the fractions are equal, the chemical shift will be exactly in the middle between the HNAM and NAM resonances. Hence at pKa $\frac{k_2}{k_1[H]} := 1$. This allows us to fix the ratio between k_1 and k_2 as

$$k_1 = k_2 10^{\mathrm{pKa}} \quad \text{(Eq. S12)}$$

Utilizing Eq. S4 and S7, the ratio from Eq. S12 and the initial condition Eq. S5, we simulated the NMR spectra as a function of pH for various rate constants k_2. Experimentally, we did not observe any visual broadening of the ^{15}N linewidth in the pH range from 2 to 12; the linewidth varied from 1.5 to 3 Hz. This was attributed to slight variations in the magnetic field during multiple days of experiments.

We were increasing the rate k_2 such that the following conditions were met, and the experimental observations were fulfilled:

1. Only one ^{15}N line is observed at all pH values in the range from pH 1 to 14.
2. The linewidth broadening due to chemical exchange can be no more than 50%.

We checked the variation of the linewidth by probing the amplitude of the spectrum and taking care that it does not go down more than 30-40%, while the integral of the spectrum remains constant. Note that amplitude is inversely proportional to line width, hence 50% increase in linewidth corresponds to a 33% decrease in amplitude. We found that these conditions are fulfilled when k_2 is equal or greater to $10^8\ s^{-1}$ (**Figure S9**). Hence, this value gives us the lower boundary for the dissociation rate. Using this rate constant, we can now estimate the effect of fast exchange on the relaxation at low magnetic fields.

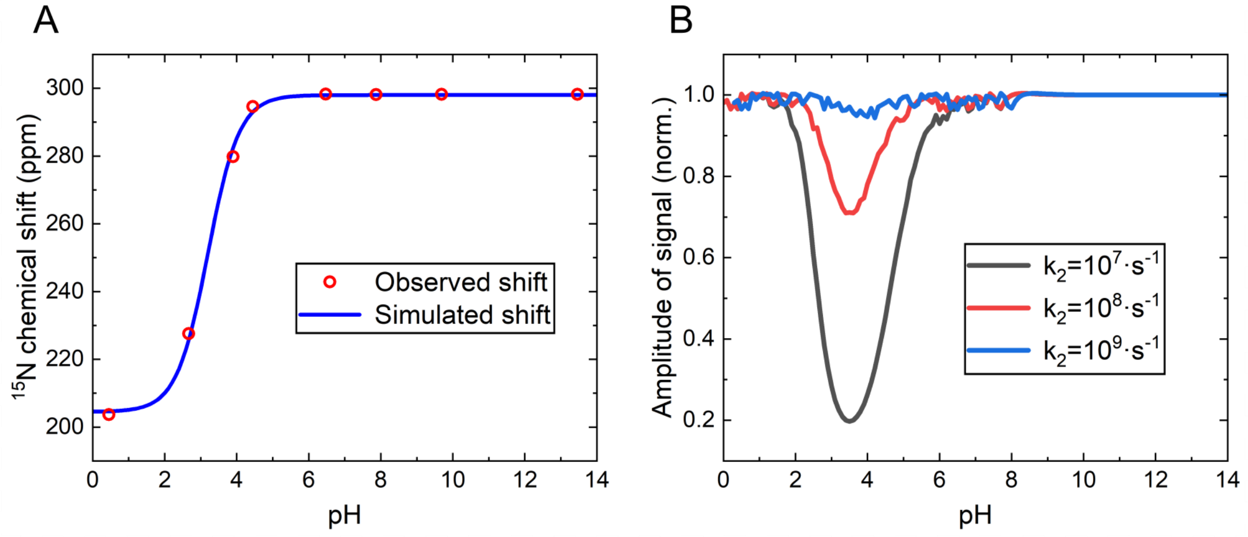

Figure S9. Chemical shifts and amplitude of the 1-^{15}N-NAM spectrum as a function of pH simulated with the Bloch-McConnell equation. These simulations (Eq. S4) allowed us (as described in the text above) to estimate the lower boundary for the hydrogen dissociation rate from HNAM. The simulation parameters were: $pKa = 3.2$, $k_2 = 10^7$, 10^8 and $10^9\ s^{-1}$, $k_1 = k_2 10^{pKa}$, Larmor precession frequency of ^{15}N $v_0 = 40\ \mathrm{MHz}$, $\delta_{NAM} = 298\ \mathrm{ppm}$, $\delta_{HNAM} = 204.5\ \mathrm{ppm}$, and $R_2 = 2\ s^{-1}$. The value $k_2 = 10^8\ s^{-1}$ appears to be good estimate for the lower boundary of the dissociation rate. In this case, less than 40% broadening of the line was observed. The total acquisition time was 5 s with 2^{16} number of points for the simulations. All amplitudes were normalized to the signal at basic pH.

10. Effects of chemical exchange on relaxation

We observed the effect of pH on the relaxation properties of 1-^{15}N-NAM. When neutral-DM is used, no polarization was observed. Experimentally, we found that neutralization can only be achieved within the stray field and a short timespan between neutralization and start of measurement.

When a slowly relaxing nucleus, here ^{15}N, has J-coupling interaction with a rapidly relaxing or rapidly (chemically) exchanging nucleus X, then the contribution of the scalar coupling relaxation as a function of a magnetic field can be calculated as follows(Ref 52, Chiavazza et al):

$$R_{1,\mathrm{SC}}^{15\mathrm{N-X}} = \frac{8\pi^2 J^2}{3} I_\mathrm{X}(I_\mathrm{X}+1)\frac{\tau_\mathrm{SC}}{1+(\omega_{15\mathrm{N}}-\omega_\mathrm{X})^2\tau_\mathrm{SC}^2} \quad \text{(Eq. S13)}$$

Here J is the constant of ^{15}N-X scalar spin-spin coupling, I_X is the spin of nucleus X (½ for ^{1}H and 1 for ^{2}H), ω_{15N} is the Larmor angular precession frequency of coupled ^{15}N and X (^{1}H or ^{2}H here) spins respectively, and τ_{SC} is the correlation time characteristic of the scalar interaction.

To estimate the T_1 value as a function of the magnetic field, we calculated the following value (**Figure S10**):

$$T_1 = \frac{1}{R_{1,\mathrm{SC}}^{15\mathrm{N-X}} + R_{10}} \quad \text{(Eq. S14)}$$

where R_{10} is the primary (here field independent) relaxation rate from different sources of relaxation. A large drop in a lifetime is predicted in these cases at low magnetic fields (**Figure S10**). Systematic measurements of T_1 as a function of pH and magnetic field have the potential to reveal the nature of the ^{15}N hyperpolarization loss.

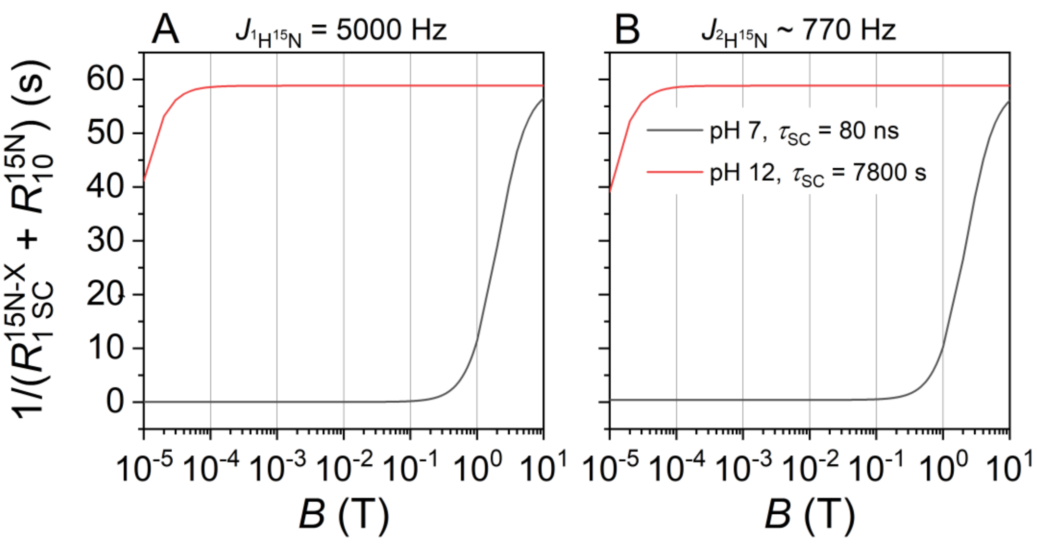

Figure S10. Effect of chemical exchange of quickly exchanging nucleus on the relaxation time of the slowly relaxing ^{15}N nucleus as a function of the magnetic field. *The interaction with ^{1}H (A) and ^{2}H (B) were considered. $R_{1,SC}$ is calculated using Eq. S14 (Ref 52, Chiavazza et al). The used parameters τ_{SC} = 80 ns (red), 7800 s (black). R_{10}^{15N} = 1/60 s^{-1}, ^{1}H-^{15}N interaction J_{1HN} = 5000 Hz, and ^{2}H-^{15}N interaction J_{2HN} = 5000/6.5 Hz were considered. This graph illustrates the effect of low magnetic fields on relaxation time in the presence of fast chemical exchange. Chemical exchanges of ^{1}H and ^{2}H from observations were compared and simulations were adjusted accordingly: no signal was found for neutral H_2O or D_2O solvents, therefore fast relaxation is expected in both cases at low fields. The correlation time was estimated as an average of NAM and HNAM lifetime: $\frac{1}{2}\left(\frac{1}{k_1[H]}+\frac{1}{k_2}\right)$ with $k_2 = 4\cdot 10^{10}\ s^{-1}$ and $k_1 = k_2 10^{pKa}$. With these parameters we can reproduce the fast relaxation at neutral pH and low magnetic fields, long relaxation time at basic pH and all magnetic fields, and relaxation time of around 10 s at neutral pH at 1 T. Such J value of 5 kHz is more in the order of dipol-dipol interaction instead of J-coupling. The estimated distance between ^{1}H and ^{15}N with a dipol-dipol interaction of J = 5 kHz can be estimated to be $r = \left(\frac{\mu_0\gamma_{1H}\gamma_{15N}\hbar^2}{4\pi hJ}\right)^{1/3}$ =1.37 Å.*

11. ^{15}N probes for 7 T MRI: nutations

Two ^{15}N-imaging probes for the clinical scanner were compared: ^{1}H,^{15}N linear surface body coils (^{1}H/^{15}N-SURF, 30 mm, O-XL-HL-070, Rapid Biomedical, **Figure S11a**) with fixed capacitors and a combination of ^{1}H quadrature whole rat body resonator (112/086 QSN, Bruker) and in-house build linear ^{15}N saddle shape coil (^{15}N-VOL, L=52 mm, ID=46 mm, **Figure S11b**).

We used a 15 mL falcon tube (L=10.7 cm, OD=1.6 cm) with 15 ml aqueous solution of 0.8 M $^{15}NH_4Cl$ with 3 vol% Gd-contrast ([Gd], 1 mmol/ml, Gadovist, Bayer) to quantify the sensitivity of the probes.

First, we studied the nutation curves for the given system (**Figure S11e,f**). The SURF coil is more power efficient than the VOL coil. The 90° signal excitation was achieved for SURF at 5.9 W, 0.27 ms, while it was at 1.9 W, 1.7 ms for VOL. The efficiency of the power use can be expressed in terms of the voltage $U=(R*P)^{½}$ times 90° duration, which is 4.6 V ms and 16.6 V ms correspondingly, meaning that VOL is about three times less efficient.

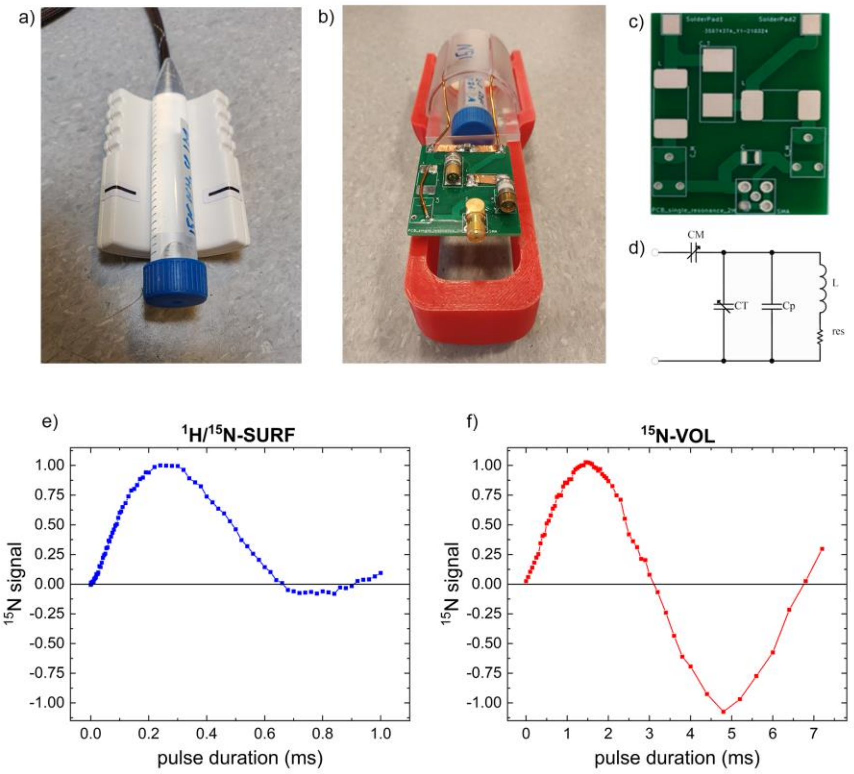

Figure S11. Comparison of commercial ^{1}H/^{15}N-SURF and home build ^{15}N-VOL coils. *Top: Images of the commercial surface coil (a, ^{1}H/^{15}N-SURF) and in-house-built linear saddle-shaped ^{15}N coil (b, ^{15}N-VOL) with the used PCB (c) and corresponding LC scheme (d). The ^{15}N-VOL inductor is made of a single 1 mm copper wire. Tuning C_T and matching C_M and offset C_p capacitors were used. Bottom: non-localized, ^{15}N MR signals of ^{1}H/^{15}N-SURF and ^{15}N-VOL of thermally polarized model solution as a function of pulse duration, revealing a maximum signal (~ 90°) at 5.9 W, 0.27 ms, and 1.9 W, 1.7 ms, respectively. Note that the signal inversion was not achieved with the surface coil.*

12. ^{15}N MRI at 7 T: thermal and hyperpolarized signals, commercial and in-house build coils

We compared the commercial and in-house coil designs and validated our method for ^{15}N imaging. The experiments were carried out for each coil design following the setup in **Figure 6**. Thermal measurements with a $^{15}NH_4Cl$-filled falcon were used to measure the sensitivity of the coils. Subsequent DNP experiments using 1-^{15}N-NAM were used for sub-1-second imaging. To quantify polarization and demonstrate spectroscopic capabilities of MRI, a single 5° spectrum was measured before image acquisition, followed by thermal polarization measurement after some delay (**Figure S12**).

Both thermal and hyperpolarized images had sufficient SNR, although SURF sensitivity was around 2.8 times higher compared to VOL. Note, however, that the VOL coil's homogeneity is superior to that of the surface coil (**Figure 6**).

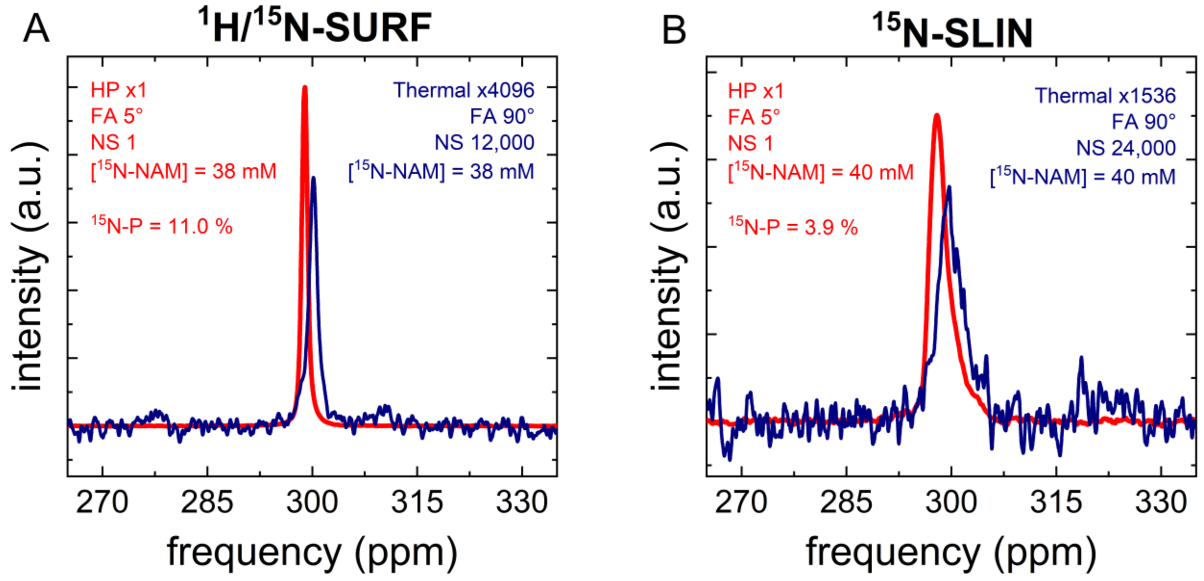

Figure S12. Thermally and hyperpolarized ^{15}N spectra. *a) Hyperpolarized (FA=5°, NS = 1, red) and thermal ^{15}N-NAM spectra (FA=90°, NS=12,000, blue). b) Hyperpolarized (FA=5°, NS = 1, red) and thermal ^{15}N-NAM spectra (FA=90°, NS=24,000, blue). The sample consisted of 204 mg of 1-^{15}N-NAM, 202 mg of H_2O, 73 mg of glycerol, and 13.3 mg of trityl radical (AH111501).*

13. Enzyme kinetic model

The enzymatic chemical reactions with NAM reported in **Figure 4** were fitted with the following equation:

$$p(t) = S_0 E_0 k \tau_e \left(1 - e^{-\frac{t}{\tau_e}}\right). \quad \text{(Eq. S15)}$$

Below we will introduce each element of this equation and demonstrate how it was derived.

We assume a one-way trimolecular reaction of the substrate (NAM) with an appropriate enzyme and co-substrate. The kinetics of this process is then given by the following differential equation:

$$\frac{d[\text{Product}]}{dt} = [\text{Substrate}][\text{Co} - \text{substrate}][\text{Enyzme}]k \quad \text{(Eq. S16)}$$

the parameter k being the conversion rate, and t is time. We assume [Substrate] and [Co-substrate] to be roughly constant due to low consumption, which we observed in the experiment (conversion of NAM to products was below 5%, **Figure 4**). However, it is reasonable to assume that the enzyme degrades during the course of the experiment, which makes [Enzyme] time dependent. When we introduce short notations p = [Product], $E(t)$ = [Enzyme], and S_0 = [Substrate]·[Co-substrates] = const, then Eq. S16 can be written as

$$\frac{dp}{dt} = S_0 E(t) k \quad \text{(Eq. S17)}$$

Unfortunately, we lack certainty about the degradation function of the enzyme. However, if we assume the typical lifetime of the enzyme to be τ_e, then a further simplification is possible:

$$E(t) = E_0 e^{-\frac{t}{\tau_e}} \quad \text{(Eq. S18)}$$

$$\frac{dp}{dt} = S_0 E_0 k e^{-\frac{t}{\tau_e}} \quad \text{(Eq. S19)}$$

Solving this equation leads to

$$p(t) = -S_0 E_0 k \tau_e e^{-\frac{t}{\tau_e}} + C_0 \quad \text{(Eq. S20)}$$

With C_0 being the integration constant. The initial conditions ($p(t = 0) = 0$ in our experiment) gives us the missing constant $C_0 = S_0 E_0 k \tau_e$, which in return gives us Eq. S15.

14. NNMT enzymatic reaction

The following preparation procedure was used to study the enzymatic reaction of NNMT (SRP6282, Sigma-Aldrich) with NAM. The experiment was carried out at 303 K and 9.4 T.

Step 1. Preparation of solutions

Prepare the following fresh solutions (A,B,C)

solution A: 5 mL H_2O with 10 mM MgCl2 + 50 mM Tris buffer + 10 mM NAM

NAM: CAS 98-92-0, Sigma-Aldrich

$MgCl_2$: CAS 7791-18-6, Sigma-Aldrich

Tris: T7943, Sigma-Aldrich

solution B: 400 µL solution A + 10 mM Co-Enzyme:

S-(5′-Adenosyl)-*L*-methioninchlorid (**SAM**),

SAM: Sigma: A7007-500mg, CAS 86867-01-8

pH was 7.84

solution C: 50 µg **hNNMT** (3.42 mg/mL)+ 50 µL D_2O for lock/reference

Right before experiment

- ☐ Put all of solution C in solution B to create solution D
- ☐ Fill solution D into NMR tube
- ☐ Shake the NMR tube for 5 s and put in NMR
- ☐ Start measurement

Final volume v = 400 µL + 50 µL + 15 µL = 465 µL.

Final concentrations (lower estimates):

[SAM] = 2.13 mg/508 g/mol/v= 9.0 mM

[NAM] = 6.36 mg/122.1 g/mol/v*400 µL/5 mL= 9.0 mM

[Tris] = 37.40 mg/149 g/mol/v*400 µL/5 mL = 43.2 mM

[MgCl] = 10.76 mg/203.3 g/mol/v*400 µL/5 mL = 9.1 mM

[**hNNMT**] added around 50 µL of the pure hNNMT.

Result:

- ☐ Conversion of NAM into 1-methyl nicotinamide 1-MNAM was observed (see main text)
- ☐ The estimated concentration of the hNNMT in the measured sample was 108 µg/mL or 32 µL/mL.
- ☐ The observed conversion was slow ($w = (2.30 \pm 0.05)\cdot 10^{-5}\ s^{-1}$) due to small enzyme amount.

15. NAMPT enzymatic reaction

The following preparation procedure was used to study the enzymatic reaction of NAMPT (SRP0514, Sigma Aldrich) with NAM. The experiment was carried out at 310 K and 9.4 T.

Step 1. Preparation of solutions

Prepare the following fresh solutions (A,B,C)

solution A: 5 mL D_2O with 10 mM $MgCl_2$ + 20 mM Phosphoribosyl pyrophosphate (PRPP) + 10 mM ATP + 50 mM Tris buffer + 10 mM NAM

NAM: CAS 98-92-0, Sigma-Aldrich

$MgCl_2$: CAS 7791-18-6, Sigma-Aldrich

Tris: T7943, Sigma-Aldrich

PRPP: CAS 108321-05-7, Sigma-Aldrich

ATP: CAS 34369-07-8, Sigma-Aldrich

solution B: 5 mL solution A + 33 µL 1:10 30% NaOH

NaOH: CAS 1310-73-2, Chemsolute

pH was 7.60

solution C: 50 µg **NAMPT** (35 µL)

Right before experiment

- ☐ Put all of solution C in 500 µL of solution B to create solution D
- ☐ Fill solution D into NMR tube
- ☐ Shake for 5 s and put in NMR
- ☐ Start measurement

Final volume v = 500 µL + 35 µL = 535 µL.

Final concentrations:

[PRPP] = 39.4 mg/390.1 g/mol/v= 17.7 mM
[ATP] = 25.1 mg/507 g/mol/v= 8.7 mM
[NAM] = 6.4 mg/122.1 g/mol/v= 9.2 mM
[Tris] = 37.5 mg/149 g/mol/v= 44.1 mM
[MgCl] = 10.74 mg/203.3 g/mol/v= 9.3 mM
[**NAMPT**] added around 35 µL of the pure NAMPT.
Result:

- ☐ Conversion of NAM into NAM-mononucleotide NMN was observed (see main text)
- ☐ The estimated concentration of the NAMPT in the measured sample was 93 µg/mL or 65 µL/mL.
- ☐ The observed conversion was slow ($w = (8.24 \pm 0.23)\cdot10^{-6}\ s^{-1}$) due to the small enzyme amount.

16. Enzymatic conversion by LDH of pyruvate

We used pure LDH enzyme to convert thermal (**Figure S13a,b**) and hyperpolarized pyruvate (**Figure S13c,d**) to lactate *in vitro*. Conversion rate of thermally polarized pyruvate to lactate using LDH was estimated to $w = (3.0 \pm 0.1) \cdot 10^{-2}\ s^{-1}$. With around 12.5 mg LDH/mL we can estimate specific activity to $k = (4.7 \pm 0.2)\ s^{-1}$ per mg/ml of enzyme and mM of pyruvate.

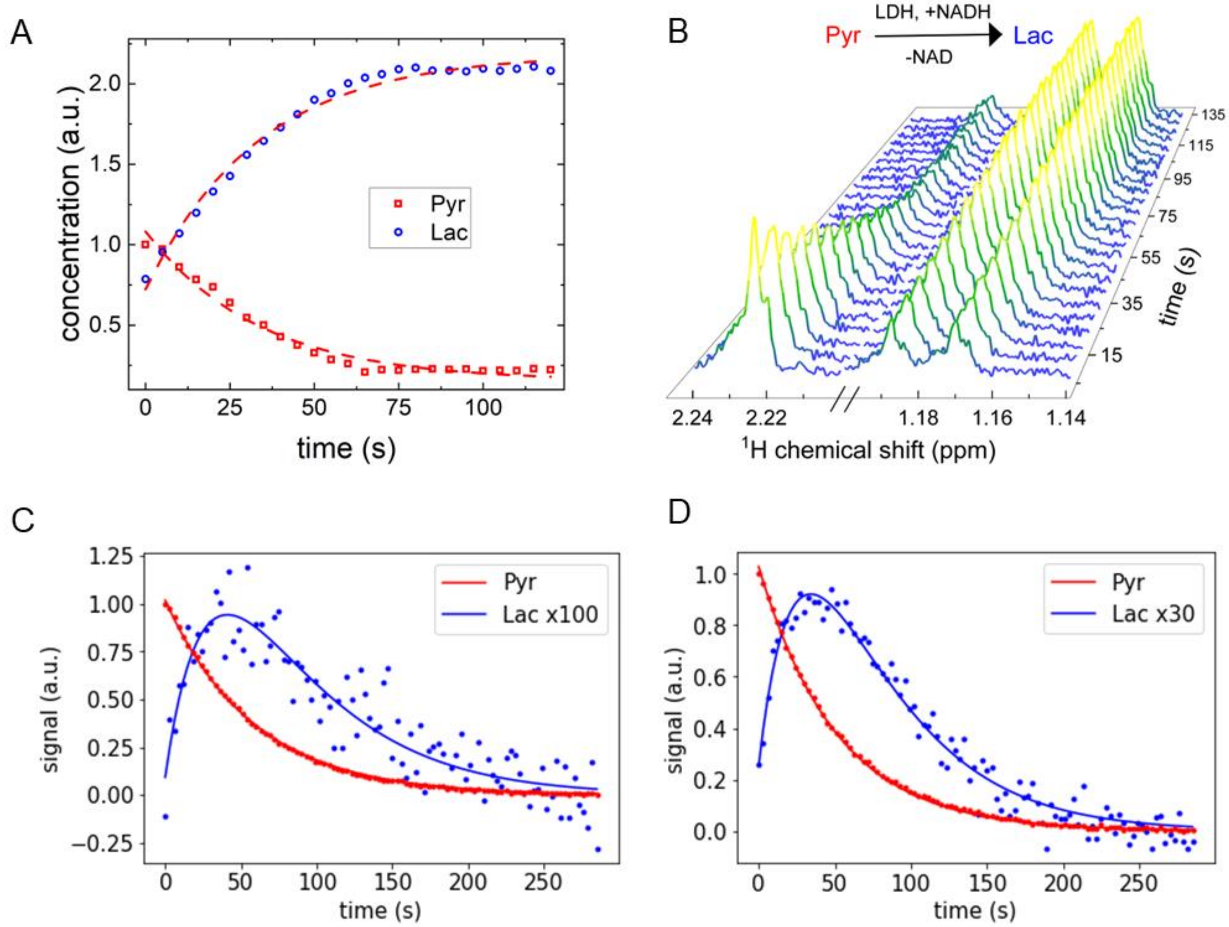

Figure S13. Thermal ^{1}H and ^{13}C hyperpolarized NMR spectra of pyruvate to lactate conversion in the presence of LDH. *(A,B) Conversion of pyruvate to lactate via the addition of 0.24 µL/mL LDH observed using ^{1}H spectroscopy. The conversion kinetics was fit (red dashed line), yielding $w = (3.0 \pm 0.1) \cdot 10^{-2}\ s^{-1}$. 2.12 mM pyruvate, 2.15 mM NADH, and PBS buffer and albumin serum were added before adding LDH as described above. (C,D) ^{13}C signals of previously hyperpolarized pyruvate (red) and lactate (blue) as a function of time. LDH and NADH induced exchange between pyruvate and lactate. Increasing the concentration of LDH resulted in an approximately linear increase in the conversion to lactate (D). Details of the experiment: FID-5° observation scheme was used with a repetition time of 3 s. Spectra were acquired using a 25 mm ^{1}H-^{13}C probe at 9.4 T. The fitted values were: T_1 relaxation times of pyruvate - 60s±5 s, lactate - 33±5 s, and exchange rate 6 $10^{-4}\ s^{-1}$ (C) and 2 $10^{-3}\ s^{-1}$ (D). The linear change of exchange rate as a function of LDH concentrations indicates that the concentration of LDH was the limiting factor.*

17. LDH conversion of pyruvate by K-562 cells

Around 2 million K-562 cells in 350 µL cell medium were mixed with 150 µL of 60 mM hyperpolarized 1-^{13}C-pyruvate. The polarization measured ~ 20 s after the dissolution was quantified to be around 27%. The sample was intensely shaken for 6 s before insertion into the NMRs isocenter. Measurements were performed every 3 s using a 5° flip angle.

This experiment showed the applicability of our experimental cell-study setup. The cells were working and metabolizing pyruvate to lactate as expected (**Figure S14**).

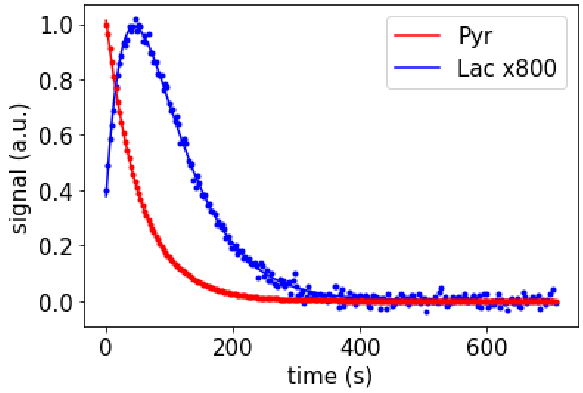

Figure S14. Graph of hyperpolarized ^{13}C pyruvate to lactate conversion in K-562 cells. *^{13}C signals of hyperpolarized pyruvate (red) and subsequently converted lactate (blue) were acquired. The K-562 cells induced the exchange between pyruvate and lactate as a result of anaerobe glycolysis. Details of the experiment: FID-5° observation scheme was used with a repetition time of 3 seconds. Spectra were acquired using a 25 mm ^{1}H-^{13}C probe at 9.4 T. The fitted values were: T_1 relaxation times of pyruvate – 57 s, lactate - 56 s, and exchange rate 5.3 10^5 s^{-1}. The conversion rate is 10 to 40 times lower than in our* in vitro *LDH experiments (**Figure S13**).*

18. Transfer magnet assembly utilizing an Halbach array

Hydrogen exchange at the 1-^{15}N site can be a cause of fast relaxation as well (Ref. 52, Chiavazza). However, utilizing 0.2-1 T Halbach magnets (**Figure S15**) during the transfer also did not improve the situation for 1-^{15}N-NAM polarization in liquid state at neutral pH.

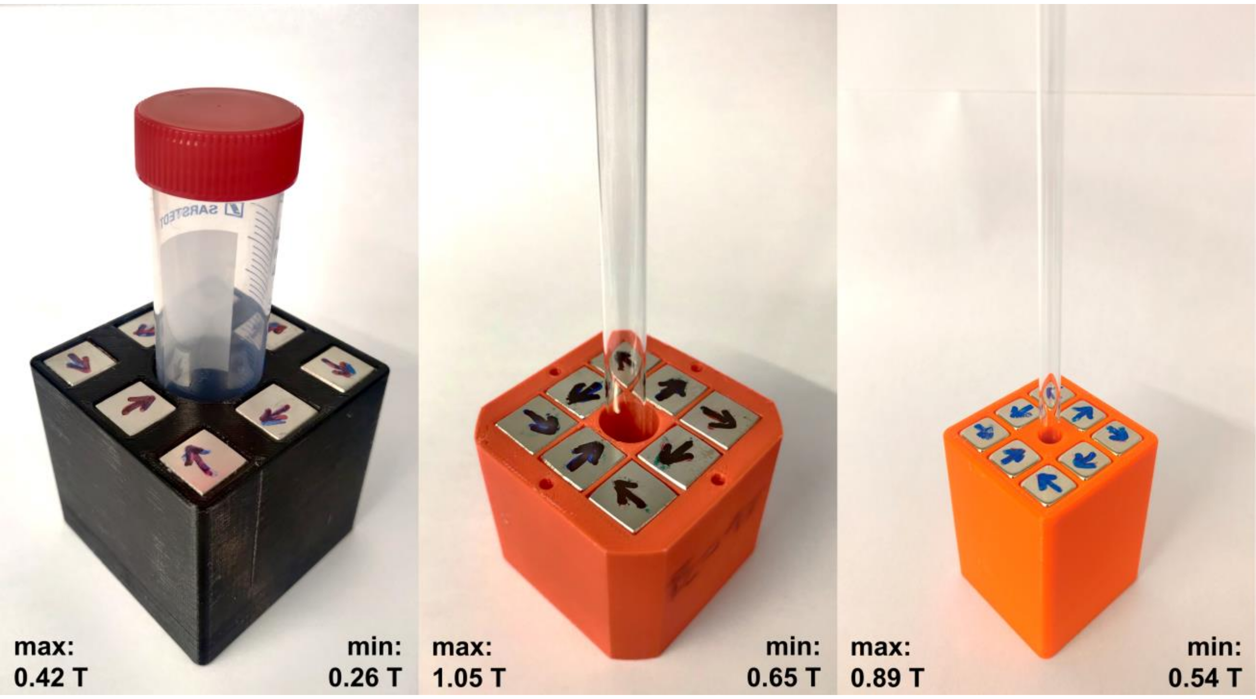

Figure S15. Vessels for transfer purposes with high-field neodymium magnets as Halbach array. The big vessel (left) was used for receiving the DNP juice after dissolution and had the lowest field-strength due to its bigger size. The other two vessels were used to transfer the DNP juice to the 0.57 T (10 mm NMR tube, middle) and 9.4 T (5 mm NMR tube, right) measuring site. All vessels were printed in house from PETG material. The orientation of neodymium bar magnets is marked by arrows. The maximum (middle of the hole) and minimum (outer edges of the hole) field strength magnitude inside the vessel is reported on the corresponding images.
